# The microbiome restrains melanoma bone growth by promoting intestinal NK and Th1 cell homing to bone

**DOI:** 10.1172/JCI157340

**Published:** 2022-06-15

**Authors:** Subhashis Pal, Daniel S. Perrien, Tetsuya Yumoto, Roberta Faccio, Andreea Stoica, Jonathan Adams, Craig M. Coopersmith, Rheinallt M. Jones, M. Neale Weitzmann, Roberto Pacifici

**Affiliations:** 1 Division of Endocrinology, Metabolism and Lipids, Department of Medicine, and; 2 Emory Microbiome Research Center, Emory University, Atlanta, Georgia, USA.; 3 Department of Surgery and Emory Critical Care Center, Emory University School of Medicine, Atlanta, Georgia, USA.; 4 Department of Orthopedics, Washington University in St. Louis, St. Louis, Missouri, USA.; 5 Division of Pediatric Gastroenterology, Hepatology, and Nutrition, Department of Pediatrics, Emory University, Atlanta, Georgia, USA.; 6 Atlanta VA Health Care System, Department of Veterans Affairs, Decatur, Georgia, USA.; 7 Immunology and Molecular Pathogenesis Program, Emory University, Atlanta, Georgia, USA.

**Keywords:** Bone Biology, Melanoma

## Abstract

Bone metastases are frequent complications of malignant melanoma leading to reduced quality of life and significant morbidity. Regulation of immune cells by the gut microbiome influences cancer progression, but the role of the microbiome in tumor growth in bone is unknown. Using intracardiac or intratibial injections of B16-F10 melanoma cells into mice, we showed that gut microbiome depletion by broad-spectrum antibiotics accelerated intraosseous tumor growth and osteolysis. Microbiome depletion blunted melanoma-induced expansion of intestinal NK cells and Th1 cells and their migration from the gut to tumor-bearing bones. Demonstrating the functional relevance of immune cell trafficking from the gut to the bone marrow (BM) in bone metastasis, blockade of S1P-mediated intestinal egress of NK and Th1 cells, or inhibition of their CXCR3/CXCL9-mediated influx into the BM, prevented the expansion of BM NK and Th1 cells and accelerated tumor growth and osteolysis. Using a mouse model, this study revealed mechanisms of microbiota-mediated gut-bone crosstalk that are relevant to the immunological restraint of melanoma metastasis and tumor growth in bone. Microbiome modifications induced by antibiotics might have negative clinical consequences in patients with melanoma.

## Graphical abstract

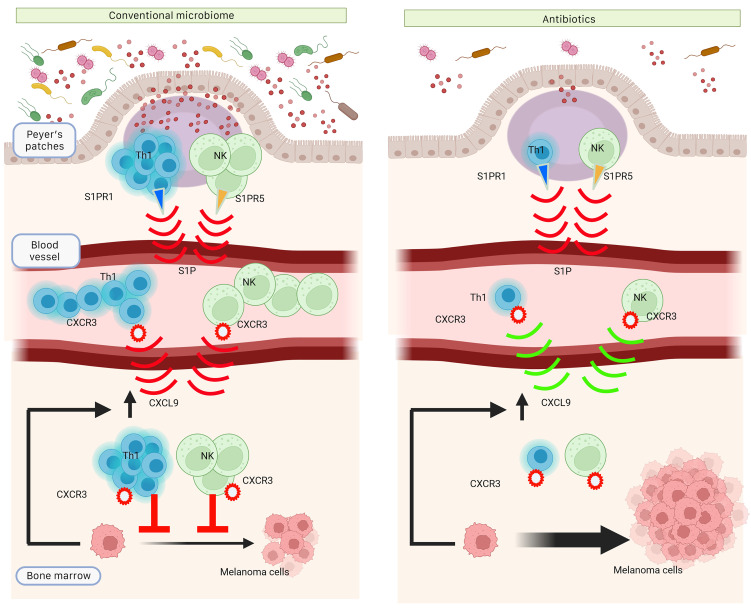

## Introduction

Microorganisms that inhabit the gut lumen are increasingly recognized as being critical to health and disease (1). One tissue now known to be regulated by the gut microbiome is bone. Studies in humans and animal models have implicated the gut microbiome as a regulator of bone mineral density (2), postnatal skeletal development (3), bone tissue material properties (4), bone mineral absorption (5), and the pathogenesis of osteoporosis (6). The microbiome is also relevant to the skeletal response to bone-regulating hormones including estrogen (7, 8), parathyroid hormone (9, 10), and glucocorticoids (11). Reports have also implicated gut microbiome composition in cancer risk (12), while a dysregulation in host cell and gut microbe interactions, a condition known as dysbiosis, has been linked to cancer progression (13, 14) and response to cancer therapeutics (15–17). However, little information is available on whether the gut microbiome plays a role in osteolytic bone lesions, which are deleterious complications of many malignancies, including malignant melanoma (18), a frequent and aggressive skin cancer (19).

The progression of bone metastasis requires the interaction between tumor cells and the tumor microenvironment, including immune cells (20). Among the immune cells involved in the response to melanoma are natural killer (NK) cells (21) and T helper 1 (Th1) cells (22). NK cells are a component of the innate immune system, accounting for nearly 10% of all peripheral lymphocytes (23). NK cells develop in the bone marrow (BM), egress into the bloodstream, and then migrate to secondary lymphoid structures including Peyer’s patches and other intestinal lymphoid tissues (24), where the local mucosal tissue microenvironment drives the acquisition of site-specific features. For example, in the gut, NK cells sense commensal bacteria and bacterial products via Toll-like receptors (TLRs) such as TLR9 (25). These interactions with the gut microbiome induce NK cell activation and cytolytic activity, which are signaled by the expression of granzyme B (GrB). The gut microbiome also promotes NK cell expression of inflammatory cytokines, and upregulates the expression of chemokine receptors that facilitate their migration to distant organs (26). Many T cell lineages are also abundant in the gut, where their differentiation, activation, and migration are regulated by the gut microbiome (27). Intestinal T cells migrate to sites of inflammation driven by chemokine gradients. For example, the gut microbiome induces the migration of TNF^+^ T cells and Th17 cells from the gut to the BM in response to an inflammatory state induced by estrogen deficiency and hyperparathyroidism (8, 9).

Additional immune cells involved in the response to cancer are cytotoxic T lymphocytes (CTLs; ref. 28), myeloid-derived suppressor cells (MDSCs; ref. 29), and mononuclear phagocytes (30). Reports have shown that the microbiome conditions the anticancer activity of these cells (31, 32). Together, this evidence suggests the possibility that the gut microbiome may contribute to the development and the progression of bone metastasis by regulating the migration of immune cells from the gut to the BM of tumor-bearing bones.

In the present study we analyzed immune cell lineages involved in the immune response to cancer and known to be regulated by the microbiome. We showed that melanoma cell growth in bone triggers expansion of NK cells and Th1 cells in the tumor-bearing bones in a gut microbiota–dependent manner. Accordingly, depletion of the gut microbiota by antibiotic treatment increases the progression of bone metastasis by blunting the migration of intestinal NK cells and Th1 cells to the BM within the bone tumor bed. Moreover, pharmacological blockade of NK or Th1 cell egress from the gut or silencing of NK and Th1 cell influx into the BM of tumor-bearing bones recapitulated the effects of microbiota depletion, preventing the expansion of these lineages in the BM and accelerating the growth of bone metastasis.

## Results

### Gut microbiota depletion by antibiotics increases tumor growth in bone by decreasing immune cells in the BM.

**Figure 1:**
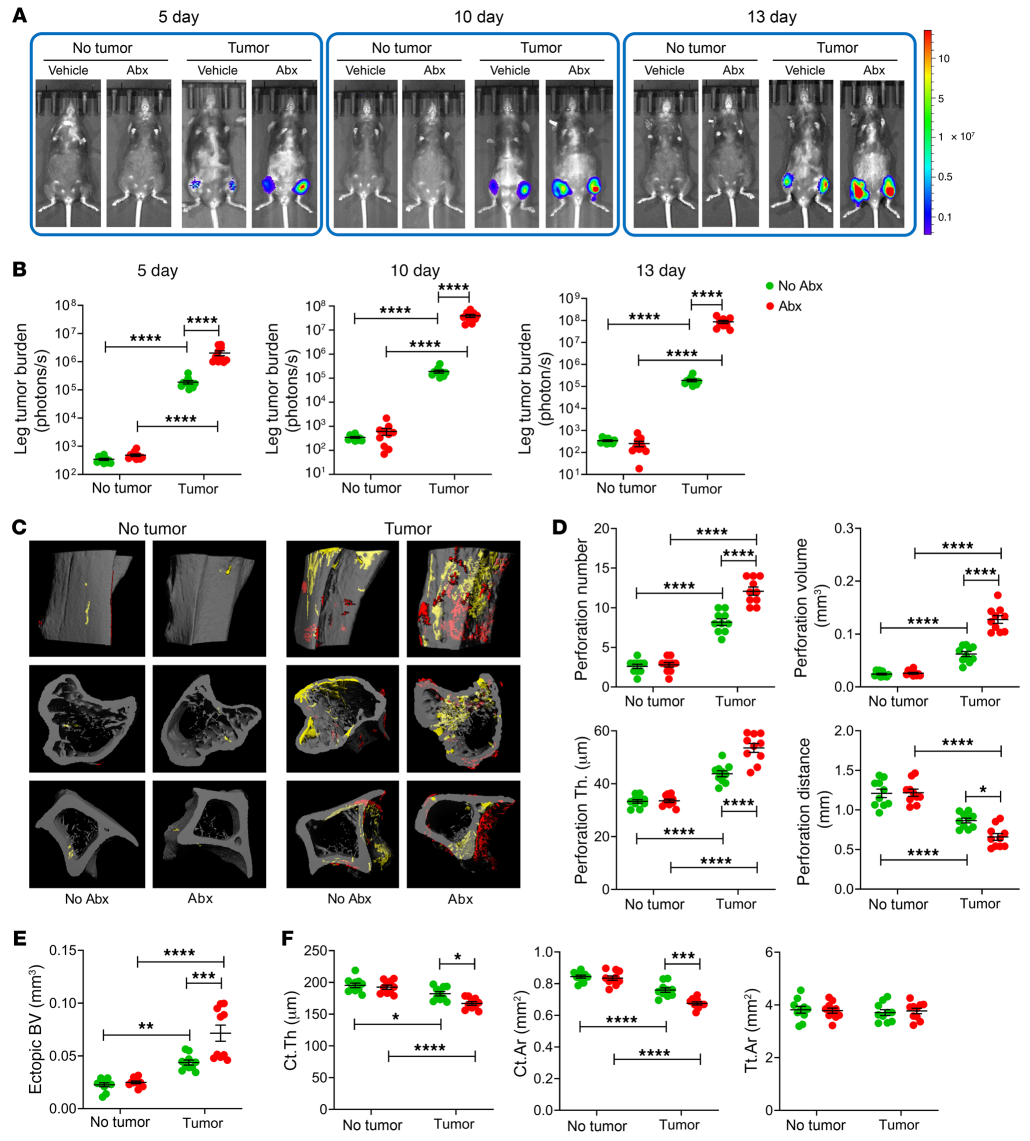
Antibiotics-induced microbiota depletion accelerates bone tumor growth caused by intracardiac injections of melanoma cells. Intracardiac injections of luciferase-expressing B16-F10 melanoma cell line were carried out in 12-week-old C57BL/6 mice. Mice not injected with B16-F10 cells (No tumor) were used as controls. Mice were treated with broad-spectrum antibiotics (1 mg/mL ampicillin, 0.5 mg/mL vancomycin, 1 mg/mL neomycin sulfate, 1 mg/mL metronidazole dissolved in water) for 4 weeks, starting 2 weeks before the tumor cell injection. (**A** and **B**) Effects of antibiotics (Abx) on tumor growth as assessed by luminescence. (**C**–**E**) Effects of Abx on bone perforations and ectopic bone growth as assessed by μCT scanning. (**C**) Representative images of the tibia. Yellow pseudocolor, perforations; red pseudocolor, ectopic bone growth. (**D** and **E**) Indices of perforation and ectopic bone formation. (**F**) μCT indices of cortical structure measured in tibial diaphysis. *n =* 10 mice per group. Data are expressed as mean ± SEM. All data were normally distributed and were analyzed by 2-way ANOVA and post hoc tests applying Bonferroni’s correction for multiple comparisons. *P < 0.05, **P < 0.01, ***P < 0.001, ****P < 0.0001 compared with the indicated group. Nonsignificant comparisons are not shown.

To investigate the extent to which the gut microbiota influences bone metastasis, intracardiac or intratibial injections of the luciferase-expressing B16-F10 melanoma cell line, a well-established mouse model of orthotopic and metastatic melanomas (33, 34), were carried out in 12-week-old C57BL/6 mice. To ablate the gut microbiome, mice were treated with broad-spectrum antibiotics (1 mg/mL ampicillin, 0.5 mg/mL vancomycin, 1 mg/mL neomycin sulfate, 1 mg/mL metronidazole administered in drinking water) for 3 weeks, starting 2 weeks before the tumor cell injection, as previously described (3, 8, 9). Analysis of fecal bacteria–specific DNA confirmed >99% ablation of the gut microbiota following antibiotic treatment, compared with non-antibiotic-treated controls (Supplemental Figure 1A; supplemental material available online with this article; https://doi.org/10.1172/JCI157340DS1). In mice subjected to intracardiac injections of B16-F10 cells, tumor growth was detected mostly in the skeleton, although at necroscopy lesions were present in the lungs, skeletal muscle, peritoneum, uterus, and ovaries. Intratibial injections led to tumor growth only at the injection site. Assessment of bone tumor growth by luminescence revealed that intracardiac injection of B16-F10 cells was followed by tumor engraftment and growth in the distal femur and proximal tibia at day 5, and further cancer growth at days 10 and 13. At each time point, bone tumor burden was greater in antibiotic-treated mice than in non-antibiotic-treated controls (Figure 1, A and B). Analysis by ex vivo micro–computed tomography (μCT) scanning of tibial proximal metaphysis harvested at day 13 revealed that injection of B16-F10 cells was followed by cortical bone perforation (pseudocolored in yellow) and ectopic bone growth (pseudocolored in red; Figure 1C). Moreover, perforation number, perforation volume, perforation thickness, and ectopic bone volume (BV) were greater in antibiotic-treated mice than in non-antibiotic-treated controls, while the distance between perforations, indicative of the nonaffected cortical bone, was shorter in antibiotic-treated than in non-antibiotic-treated mice (Figure 1, D and E). At the tibial midshaft, an area devoid of perforations, a decrease in cortical thickness (Ct.Th) and cortical area (Ct.Ar) with no changes in total area (Tt.Ar) was also detected, indicating that B16-F10 cell injection caused cortical bone loss. The changes in these indices of cortical structure were greater in antibiotic-treated tumor-bearing mice than in non-antibiotic-treated tumor-bearing controls (Figure 1F). In addition, metaphyseal trabecular BV, trabecular bone volume fraction (BV/TV), trabecular thickness (Tb.Th), trabecular number (Tb.N), and trabecular separation (Tb.Sp), which are indices of trabecular structure, were all affected by cancer cell injection (Supplemental Figure 2A). Moreover, in mice injected with cancer cells, antibiotic treatment resulted in greater alterations of BV/TV and Tb.Th, in comparison with non-antibiotic-treated controls.

**Figure 2:**
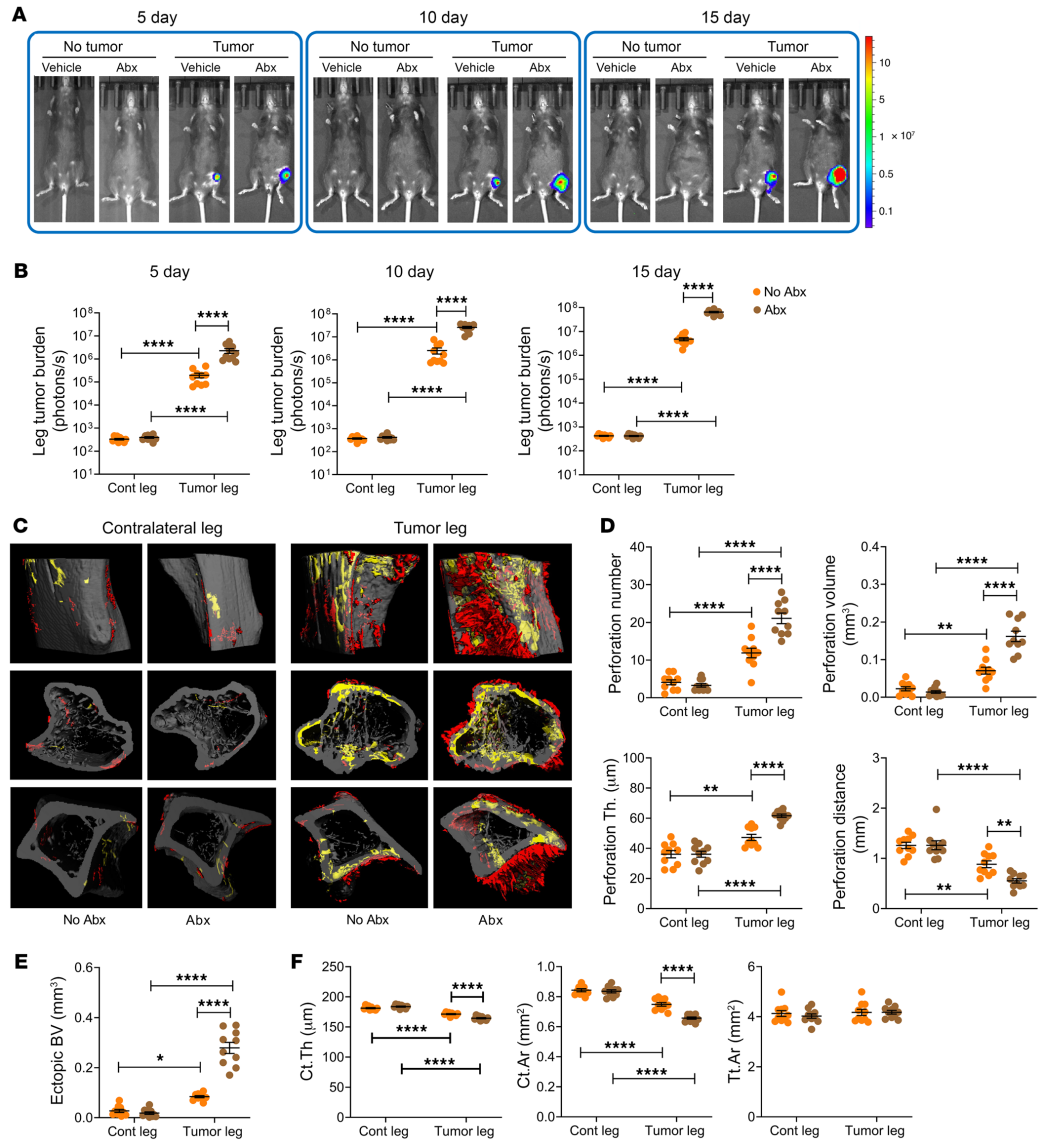
Antibiotics-induced microbiota depletion accelerates bone tumor growth caused by intratibial injections of melanoma cells. Intratibial injections of luciferase-expressing B16-F10 melanoma cell line were carried out in 12-week-old C57BL/6 mice. The noninjected contralateral leg (Cont leg) was used as a control. Mice were treated with broad-spectrum antibiotics for 4 weeks, starting 2 weeks before the tumor cell injection. (**A** and **B**) Effects of antibiotics on tumor growth as assessed by luminescence. (**C**–**E**) Effects of antibiotics on bone perforations and ectopic bone growth as assessed by μCT. (**C**) Representative images of the tibia. Yellow pseudocolor, perforations; red pseudocolor, ectopic bone growth. (**D** and **E**) Indices of perforation and ectopic bone formation. (**F**) μCT indices of cortical structure measured in tibial diaphysis. *n =* 10 mice per group. Data are expressed as mean ± SEM. All data were normally distributed and were analyzed by 2-way ANOVA and post hoc tests applying Bonferroni’s correction for multiple comparisons. *P < 0.05, **P < 0.01, ****P < 0.0001 compared with the indicated group. Nonsignificant comparisons are not shown.

To corroborate these studies, we used a further experimental model, the intratibial injection of B16-F10 cells. Following intratibial B16-F10 cell injection, tumor growth peaked at day 15. At each time point measured, tumor burden was greater in antibiotic-treated mice than in non-antibiotic-treated controls (Figure 2, A and B). μCT analysis of tibiae harvested at day 15 revealed tumor-induced cortical perforations and ectopic bone growth in the proximal metaphysis. These lesions were greater in tumor-injected bones of antibiotic-treated mice compared with the tumor-injected legs of non-antibiotic-treated mice (Figure 2, C–E). Midshaft Ct.Th and Ct.Ar were decreased by tumor growth in all groups, but among the tumor-injected mice, cortical bone loss was more severe in antibiotic-treated mice than in non-antibiotic-treated controls (Figure 2F). These data suggest that in mice subjected to the intratibial injection of B16-F10 cells, microbiota depletion by antibiotic treatment increased tumor burden and worsened tumor-induced osteolysis and ectopic bone formation. All indices of trabecular volume and structure in the proximal metaphysis were negatively affected by cancer cell injection. These alterations were significantly exacerbated by antibiotics (Supplemental Figure 2B).

To confirm that the effects of broad-spectrum antibiotics on bone tumor burden and osteolysis arose from direct effects of antibiotics on the intestinal bacteria, rather than from off-target effects of antibiotics, mice were treated with 2 nonabsorbable antibiotics (2 mg/mL neomycin sulfate, 2 mg/mL bacitracin dissolved in drinking water) for the duration of the experiment, starting 2 weeks before the tumor cell injection. The effectiveness of nonabsorbable antibiotics was similar to that of broad-spectrum antibiotics, as they ablated the gut microbiota by >99%, compared with controls (Supplemental Figure 1B). Intracardiac injection of B16-F10 cells was followed by bone tumor growth that peaked at day 13. At each time point, tumor burden was greater in mice treated with nonabsorbable antibiotics than in non-antibiotic-treated controls (Supplemental Figure 3, A and B). μCT analysis of tibiae revealed that injection of B16-F10 cells led to cortical bone perforation and ectopic bone growth. The number of perforations, perforation volume, perforation thickness, and ectopic bone volume were greater in mice treated with nonabsorbable antibiotics than in non-antibiotic-treated controls (Supplemental Figure 3, C–E). The distance between perforations and midshaft, Ct.Ar, and Ct.Th were lower in nonabsorbable antibiotic–treated mice than in nontreated controls (Supplemental Figure 3F). In addition, indices of trabecular structure were all affected by cancer cell injection. Alterations of these indices were exacerbated by antibiotics (Supplemental Figure 4A).

Nonabsorbable antibiotics were also administered to mice subjected to intratibial injection of B16-F10 cells. In these experiments, local tumor growth peaked at day 15. At each time point, bone tumor burden was greater in nonabsorbable antibiotic–treated mice than in untreated controls (Supplemental Figure 5, A and B). μCT analysis of tibiae harvested at day 15 revealed that cancer cells caused cortical bone perforation and ectopic bone growth. Perforation indices and ectopic bone volume were more affected in nonabsorbable antibiotic–treated mice than in non-antibiotic-treated controls (Supplemental Figure 5, C–E). Midshaft Ct.Ar and Ct.Th were more significantly decreased by cancer growth in antibiotic-treated mice than in non-antibiotic-treated mice (Supplemental Figure 5F). Indices of trabecular structure were all affected by cancer cell injection and further exacerbated in antibiotic-treated mice (Supplemental Figure 4B). Since nonabsorbable antibiotics and broad-spectrum antibiotics were both effective in altering the measured indices, it is unlikely that the activity of antibiotics is due to an off-target effect of these agents.

### Gut microbiota depletion by antibiotics impacts NK and Th1 cell frequency in the BM.

**Figure 3:**
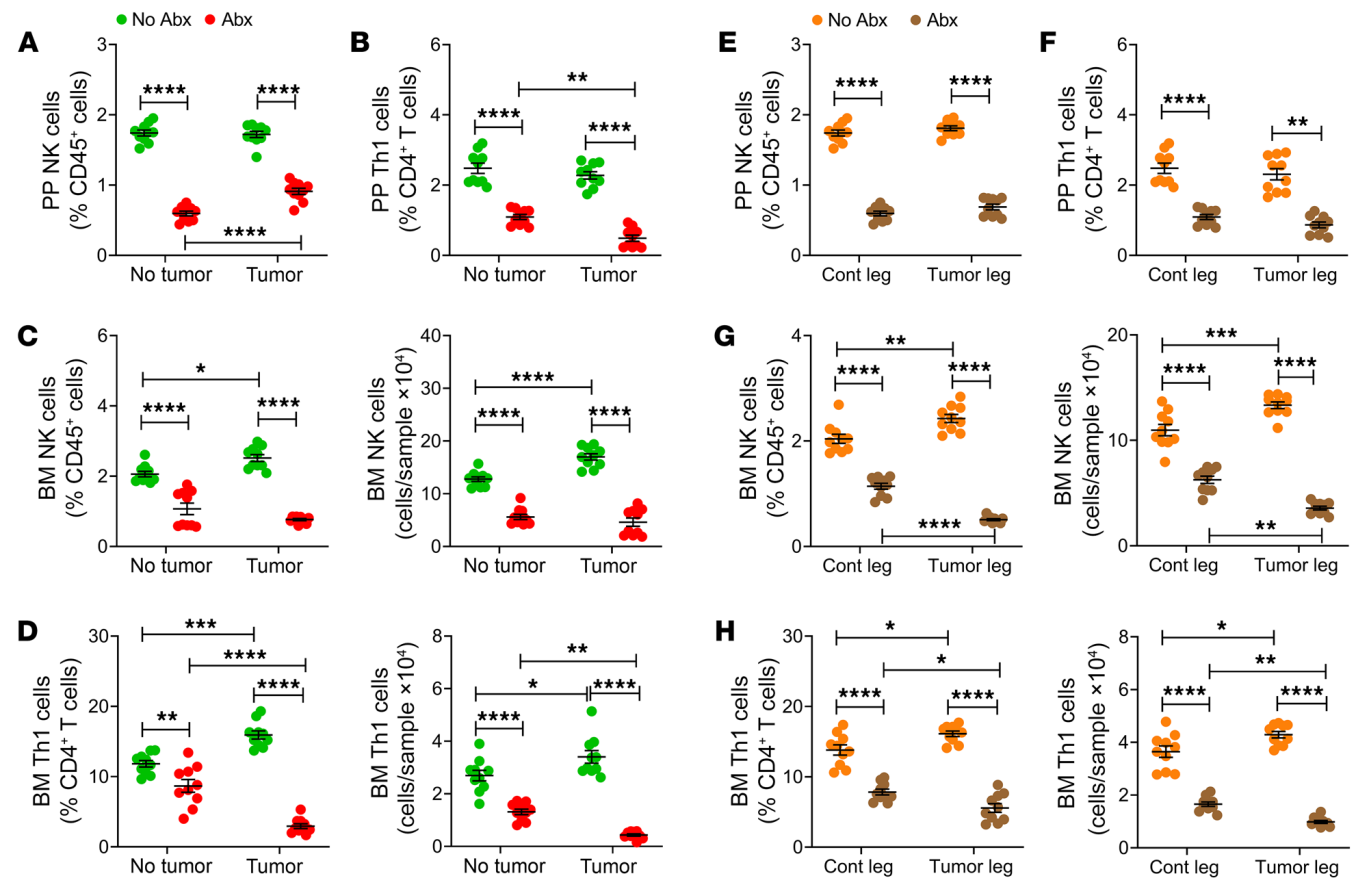
Antibiotics-induced microbiota depletion blocks the expansion of BM NK cells and Th1 cells caused by intracardiac and intratibial injection of melanoma cells. Intracardiac or intratibial injections of luciferase-expressing B16-F10 melanoma cell line were carried out in 12-week-old C57BL/6 mice. In the intracardiac model, mice not injected with B16-F10 cells (No tumor) were used as controls. In the intratibial model, the noninjected contralateral leg (Cont leg) was used as control. Mice were treated with broad-spectrum antibiotics for 4 weeks, starting 2 weeks before the tumor cell injection. (**A** and **E**) Relative frequency of PP NK (NK1.1^+^CD3^+^) cells. (**B** and **F**) Relative frequency of PP Th1 (CD3^+^CD4^+^IFN-γ^+^) cells. (**C** and **G**) Relative and absolute frequency of BM NK cells. (**D** and **H**) Relative and absolute frequency of BM Th1 cells. (**A**–**D**) Intracardiac injections. (**E**–**H**) Intratibial injections. *n =* 10 mice per group. Data are expressed as mean ± SEM. All data were normally distributed and were analyzed by 2-way ANOVA and post hoc tests applying Bonferroni’s correction for multiple comparisons. *P < 0.05, **P < 0.01, ***P < 0.001, ****P < 0.0001 compared with the indicated group. Nonsignificant comparisons are not shown.

Little information is available on the effects of the microbiome on NK cells and Th1 cells. To investigate this, the frequency of NK cells (CD45^+^CD3^–^NK1.1^+^ cells) and Th1 cells (CD3^+^TCRβ^+^CD4^+^IFN-γ^+^ cells) in the Peyer’s patches (PPs) and in the BM was determined by flow cytometry (Supplemental Figure 6) using samples harvested at sacrifice. Because the measurement of the absolute number of PP cells is technically challenging owing to variability of the size of the collected PP tissue, NK cells and Th1 cells in PPs were quantified as percentage of total cells. In non-antibiotic-treated mice, intracardiac injection of B16-F10 cells caused no changes in PP NK and Th1 cell frequencies (Figure 3, A and B). By contrast, the frequency of both NK cells and Th1 cells increased in response to tumor cell injection in the BM (Figure 3, C and D). Broad-spectrum antibiotics lowered the frequency of PP and BM NK cells and Th1 cells in healthy controls and in cancer cell–injected mice (Figure 3, A–D). As a result, while intracardiac melanoma cell injection increased the frequency of BM NK cells and Th1 cells in non-antibiotic-treated controls, it did not affect the frequency of BM NK cells and decreased the frequency of BM Th1 cells in antibiotic-treated mice (Figure 3, A–D). These data demonstrated a role for the gut microbiome in the changes in immune response to cancer cells in bone.

Studies conducted using the intratibial injection of B16-F10 cells confirmed these findings. In fact, intratibial melanoma cell injection did not affect the frequency of PP NK cells and Th1 cells (Figure 3, E and F) but increased the frequency of NK cells and Th1 cells within the tumor-bearing bones of mice not treated with antibiotics (Figure 3, G and H). Moreover, antibiotic treatment lowered the frequency of PP and BM NK cells and Th1 cells in healthy controls and in cancer cell–injected mice (Figure 3, E–H).

Analysis of CD8^+^ T cells, cytolytic CD8^+^ T cells (GrB^+^ CD8^+^ T cells), polymorphonucleated MDSCs, and monocytic MDSCs — cell populations involved in immune responses to cancer — revealed that microbiome depletion by antibiotics decreased the relative frequency of most of these populations in PPs (Supplemental Figure 7). By contrast, antibiotics either did not affect or increased the absolute frequency of CD8^+^ T cells, GrB^+^ CD8^+^ T cells, polymorphonucleated MDSCs, and monocytic MDSCs in the BM (Supplemental Figure 7). The finding of discordant effects of antibiotics on the analyzed cell lineages in PPs and BM indicated that trafficking of intestinal CD8^+^ T cells and MDSCs to the BM does not contribute to the cancer cell–induced expansion of these lineages in the BM.

Intracardiac injection of B16-F10 cells in mice treated with nonabsorbable antibiotics did not change the frequency of NK cells, GrB-expressing (GrB^+^) activated NK cells, and Th1 cells in PPs (Supplemental Figure 8A). However, NK cells, GrB^+^ NK cells, and Th1 cells in BM increased in response to tumor cell injection (Supplemental Figure 8B). Nonabsorbable antibiotic treatment lowered the frequency of PP and BM NK cells, GrB^+^ NK cells, and Th1 cells in healthy controls and in cancer cell–injected mice (Supplemental Figure 8, A and B), confirming the results of the broad-spectrum antibiotic experiment. Similarly, intratibial injection of B16-F10 cells did not affect the frequency of PP NK cells, GrB^+^ NK cells, and Th1 cells (Supplemental Figure 8C) but did increase the frequency of these lineages in the BM (Supplemental Figure 8D). Moreover, antibiotic treatment lowered the frequency of PP and BM NK cells, GrB^+^ NK cells, and Th1 cells in healthy controls and in cancer cell–injected mice (Supplemental Figure 8, C and D). Together, these findings indicate that the capacity of antibiotics to regulate NK cells and Th1 cells does not result from an off-target effect of these agents.

### B16-F10 cancer cells promote the migration of intestinal immune cells to the BM via a microbiota-dependent effect.

Since intestinal immune cells are known to migrate to distant organs, bone tumor growth may cause the homing of these immune cells to the affected bones. Whether this is mediated by direct effects of the tumor or indirect responses of the BM cells to the tumor is presently unknown.

To directly investigate the effect of cancer bone growth on NK cells and Th1 cell trafficking, we utilized C57BL/6 Kaede mice (35) using methods established in our laboratory (8). This mouse strain offers a sensitive means of tracking the migration from the gut to anatomically distant sites of any leukocyte population definable by surface-displayed or intracellular markers. Kaede mice ubiquitously express the photoconvertible protein Kaede, which permanently changes its fluorescence emission from green (518 nm) to red (582 nm) upon photoactivation with near-UV light (350–410 nm). Once photoconverted in the intestine, red-fluorescing cells can be detected and enumerated by flow cytometry in other organs. The photoconversion of intracellular Kaede has no effect on cellular function and on the homing capacity of T cells (36). Hereafter, we will refer to photoconverted cells as KaedeR cells. Supplemental Figure 9, A and B, shows the intestine and fluorescence microscopy images of PP cells of a Kaede mouse subjected or not subjected to photoactivation of the entire dissected organ ex vivo by exposure to a 390 nm light for 2 minutes. Intracardiac or intratibial injections of B16-F10 cells were performed in 12-week-old Kaede mice. Nine days later, when bone lesions were well established and rapidly growing, all animals were subjected to surgical laparotomy, and 4 PPs per mouse were photoactivated by exposure to a 390 nm light for 2 minutes. To ensure that no other cells were photoconverted, the whole mouse was covered with an aluminum foil blanket. Mice were sacrificed 24 or 48 hours later, and the number of KaedeR NK cells, GrB^+^ NK cells, and Th1 cells in PPs and BM was measured by flow cytometry.

**Figure 4:**
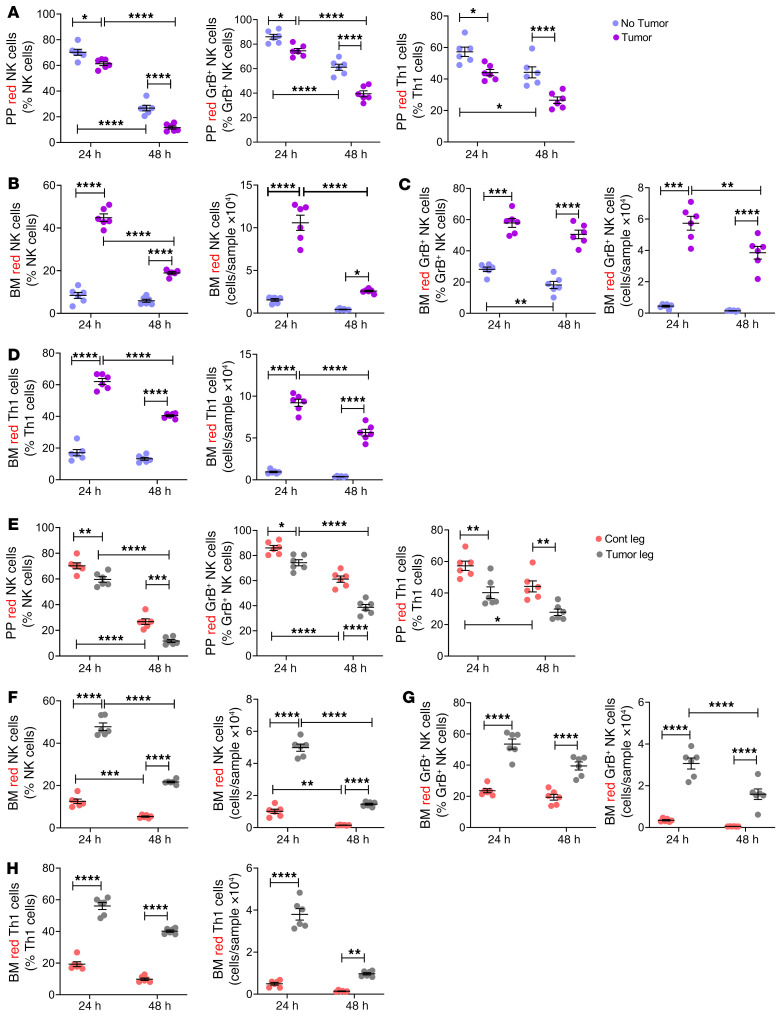
Bone tumor growth increases trafficking of NK cells and Th1 cells from the gut to the BM within the tumor lesion. Intracardiac (**A**–**D**) or intratibial injections (**E**–**H**) of B16-F10 cells were carried out in 12-week-old Kaede mice. In the intracardiac model, mice not injected with B16-F10 cells (No tumor) were used as controls. In the intratibial model, the noninjected contralateral leg (Cont leg) was used as control. Nine days later, mice were subjected to surgical laparotomy to access the PPs in the distal SI. PP cells were photoconverted by exposure to a 390 nm light for 2 minutes. Mice were sacrificed 24 and 48 hours after photoconversion. (**A** and **E**) Relative frequency of PP NK (NK1.1^+^CD3^+^) cells, GrB^+^ NK cells, and Th1 (CD3^+^CD4^+^IFN-γ^+^) cells. (**B** and **F**) Relative and absolute frequency of BM NK cells. (**C** and **G**) Relative and absolute frequency of BM GrB^+^ NK cells. (**D** and **H**) Relative and absolute frequency of BM Th1 cells. *n =* 6 mice per group. Data are expressed as mean ± SEM. All data were normally distributed and were analyzed by 2-way ANOVA and post hoc tests applying Bonferroni’s correction for multiple comparisons. *P < 0.05, **P < 0.01, ***P < 0.001, ****P < 0.0001 compared with the indicated group. Nonsignificant comparisons are not shown.

Intracardiac and intratibial injection of B16-F10 cells was followed by a decrease in the frequency of PP KaedeR NK cells, GrB^+^ NK cells (37), and Th1 cells at 24 and 48 hours with a peak at 48 hours (Figure 4, A and E, and Supplemental Figure 9C). These findings indicated that the egress of NK cells, GrB^+^ NK cells, and Th1 cells from PPs increased during bone cancer growth. Analysis of BM cells from healthy mice revealed that approximately 10%–30% of the BM NK cells, GrB^+^ NK cells, and Th1 cells were KaedeR T cells (Figure 4, B–D and F–H, and Supplemental Figure 9D). This was consistent with the fact that, since the cells of 4 PPs per mouse had been photoconverted, a large fraction of the intestinal immune cells with the potential to migrate to the BM were Kaede red cells. Injection of B16-F10 cells markedly increased the relative and absolute frequency of BM NK cells, GrB^+^ NK cells, and Th1 cells at 24 and 48 hours with a peak at 45%–60% at 24 hours (Figure 4, B–D and F–H). This effect was site specific, since intracardiac or intratibial injection of B16-F10 cells into Kaede mice did not increase the migration of KaedeR NK cells, GrB^+^ NK cells, and Th1 cells to the spleen or the liver (Supplemental Figure 10, A and B), tissues where cancer cells were not detected at necroscopy. Together, these data demonstrate that intraosseous melanoma tumor growth promotes the migration of intestinal NK cells, GrB^+^ NK cells, and Th1 cells from intestinal tissues to bone.

**Figure 5:**
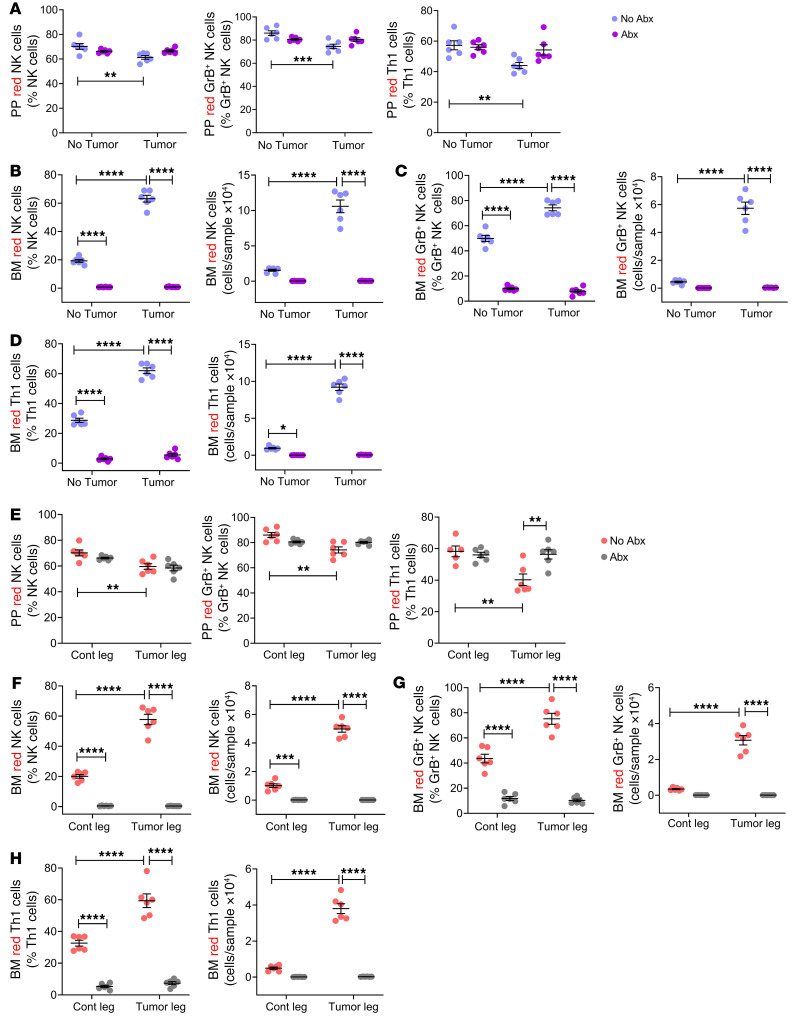
Antibiotics-induced microbiota depletion prevents trafficking of NK cells and Th1 cells from the gut to the BM induced by tumor growth. Intracardiac (**A**–**D**) or intratibial injections (**E**–**H**) of B16-F10 cells were carried out in 12-week-old Kaede mice treated or not treated with broad-spectrum antibiotics. In the intracardiac model, mice not injected with B16-F10 cells (No tumor) were used as controls. In the intratibial model, the noninjected contralateral leg (Cont leg) was used as control. Nine days later, mice were subjected to surgical laparotomy to access the PPs in the distal SI. PP cells were photoconverted by exposure to a 390 nm light for 2 minutes. Mice were sacrificed 24 hours after photoconversion. (**A** and **E**) Relative frequency of PP NK (NK1.1^+^CD3^+^) cells, GrB^+^ NK cells, and Th1 (CD3^+^CD4^+^IFN-γ^+^) cells. (**B** and **F**) Relative and absolute frequency of BM NK cells. (**C** and **G**) Relative and absolute frequency of BM GrB^+^ NK cells. (**D** and **H**) Relative and absolute frequency of BM Th1 cells. *n =* 6 mice per group. Data are expressed as mean ± SEM. All data were normally distributed and were analyzed by 2-way ANOVA and post hoc tests applying Bonferroni’s correction for multiple comparisons. *P < 0.05, **P < 0.01, ***P < 0.001, ****P < 0.0001 compared with the indicated group. Nonsignificant comparisons are not shown.

To determine whether the migration of immune cells from the gut to the BM was microbiota dependent, Kaede mice were subjected to intracardiac or intratibial injection of B16-F10 cells. Some groups of mice were treated with broad-spectrum antibiotics for 3 weeks, starting 2 weeks before the tumor cell injection. Nine days after the tumor cell injection, all animals were subjected to laparotomy, and 4 PPs per mouse were photoactivated. Mice were sacrificed 24 hours later, and the number of KaedeR NK cells, GrB^+^ NK cells, and Th1 cells in PPs and BM was measured by flow cytometry. In both the intracardiac (Figure 5, A–D) and the intratibial experimental model (Figure 5, E–H), antibiotics prevented the decrease in the frequency of PP KaedeR NK cells, GrB^+^ NK cells, and Th1 cells induced by injection of B16-F10 cells (Figure 5, A and E). Antibiotics also completely prevented the increase in the frequency of these cells in the BM (Figure 5, B–D and F–H), demonstrating that the melanoma-induced increase in the migration of PP NK cells, GrB^+^ NK cells, and Th1 cells induced by B16-F10 cells was microbiota dependent.

### S1PR1 and S1PR5 induce the egress of Th1 and NK1 cells from the small intestine.

**Figure 6:**
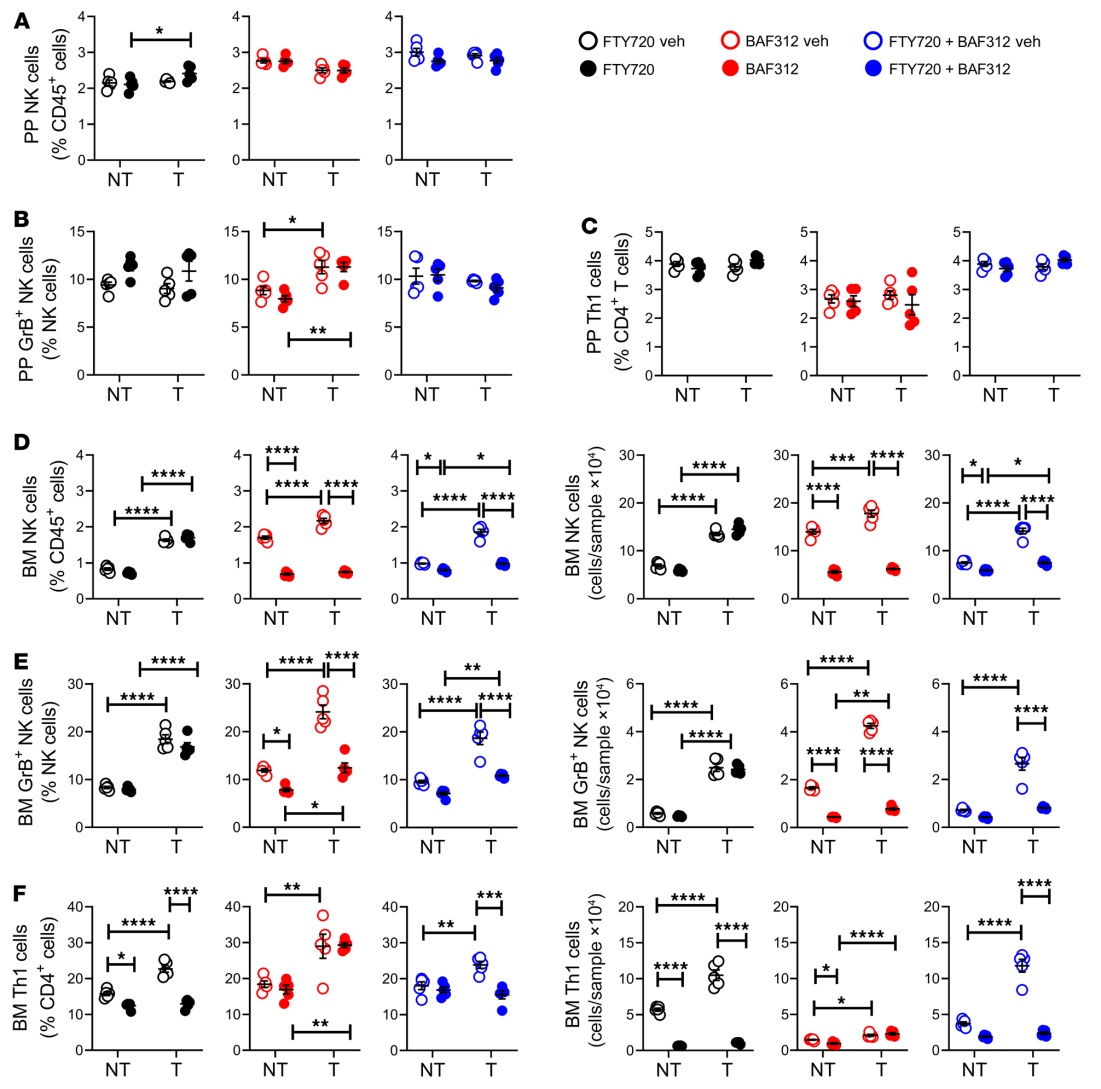
Blockade of Th1 and NK cell egress from the gut prevents the expansion of BM Th1 and NK cells induced by intracardiac injections of melanoma cells. Intracardiac injections of luciferase-expressing B16-F10 melanoma cell line were carried out in 12-week-old C57BL/6 mice. Mice not injected with B16-F10 cells (No tumor [NT]) were used as controls. Mice were also treated with the S1PR1 functional antagonist FTY720 and/or the S1PR5 functional antagonist BAF312, starting 1 week before the tumor cell injection. (**A**) Relative frequency of PP NK (NK1.1^+^CD3^+^) cells. (**B**) Relative frequency of PP GrB^+^ NK cells. (**C**) Relative frequency of PP Th1 (CD3^+^CD4^+^IFN-γ^+^) cells. (**D**) Relative and absolute frequency of BM NK cells. (**E**) Relative and absolute frequency of BM GrB^+^ NK cells. (**F**) Relative and absolute frequency of BM Th1 cells. *n =* 5 mice per group. Data are expressed as mean ± SEM. All data were normally distributed and were analyzed by 2-way ANOVA and post hoc tests applying Bonferroni’s correction for multiple comparisons. *P < 0.05, **P < 0.01, ***P < 0.001, ****P < 0.0001 compared with the indicated group. Nonsignificant comparisons are not shown. T, tumor.

The chemokine receptors S1PR1 and S1PR5 are expressed by T cells and NK cells, respectively (38, 39). These receptors promote the egress of intestinal T cells and NK cells in response to sensing of circulating S1P (38). To investigate the hypothesis that melanoma growth in bone promotes the egress of Th1 cells and NK cells from the intestine through an S1PR1/5-mediated mechanism, animals were injected with B16-F10 cells and treated for 3 weeks with the S1PR1 functional antagonist FTY720, the S1PR5 functional antagonist BAF312, or both FTY720 and BAF312. These agents arrest the exit of lymphocytes or NK cells from PPs and mesenteric lymph nodes (40, 41) without affecting their function (40–43). In both the intracardiac and intratibial B16-F10 cell injection models, treatment with FTY720 and/or BAF312 had no effects on the frequency of PP NK cells, GrB^+^ NK cells, and Th1 cells, in either control or tumor cell–injected mice (Figure 6, A–C, and Supplemental Figure 11, A–C). Moreover, FTY720 did not affect the increase in BM NK cells and GrB^+^ NK cells induced by cancer growth (Figure 6, D and E, and Supplemental Figure 11, D and E), while it prevented the tumor-induced increase of Th1 cells in the BM (Figure 6F and Supplemental Figure 11F). These findings showed that blockade of the egress of Th1 cells from the gut blunts the expansion of these cells in BM via an S1PR1-mediated mechanism. By contrast, treatment with BAF312 prevented the tumor-induced increase of NK cells and GrB^+^ NK cells in the BM (Figure 6, D and E, and Supplemental Figure 11, D and E) but did not affect the frequency of BM Th1 cells (Figure 6F and Supplemental Figure 11F), demonstrating that S1PR5 signaling mediates the egress of NK cells from the gut and their homing to the BM. In agreement with this hypothesis, treatment with both FTY720 and BAF312 prevented the tumor-induced increase of NK cells, GrB^+^ NK cells, andTh1 cells in the BM (Figure 6, D–F, and Supplemental Figure 11, D–F).

**Figure 7:**
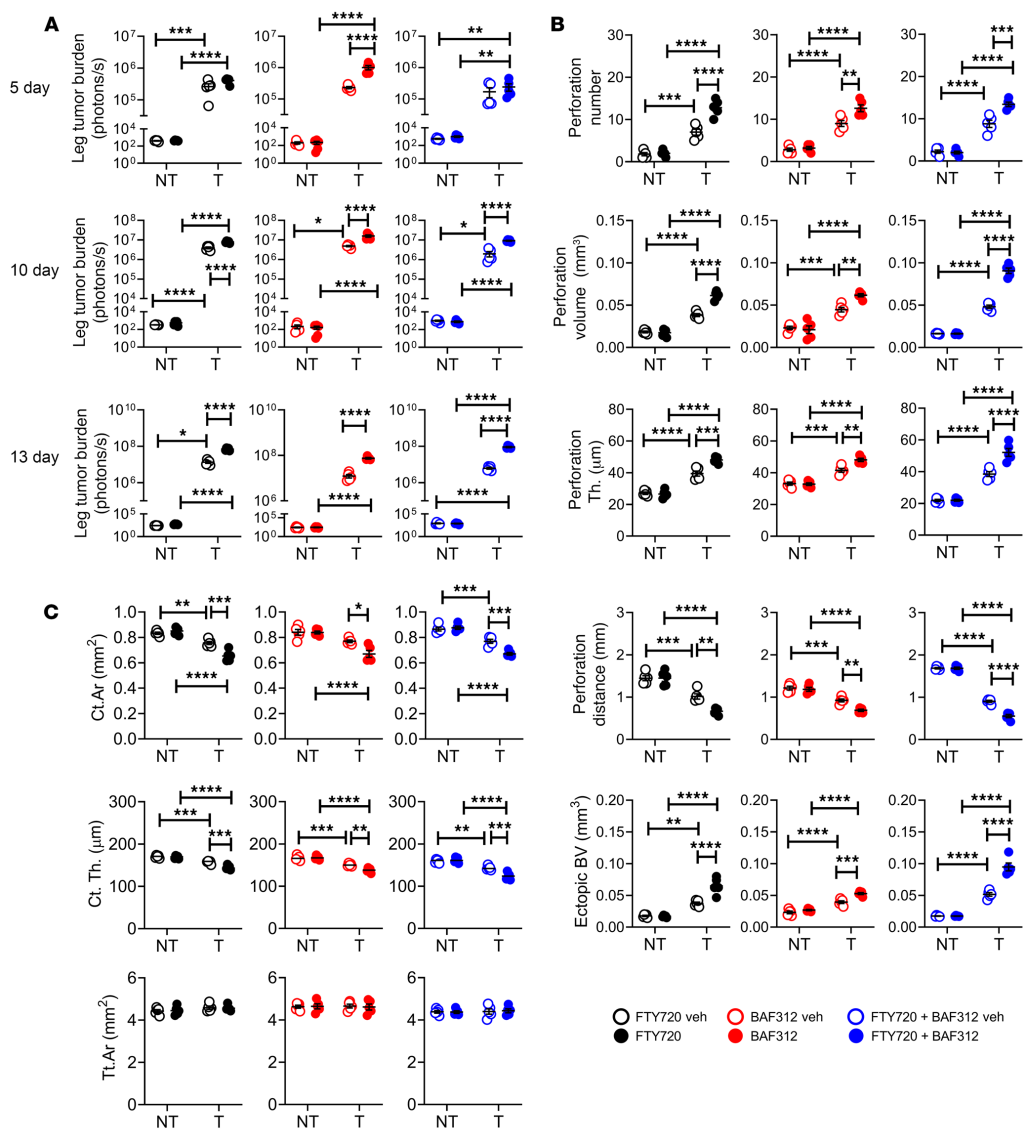
Blockade of Th1 and NK cell egress from the gut accelerates bone tumor growth induced by intracardiac injections of melanoma cells. Intracardiac injections of luciferase-expressing B16-F10 melanoma cell line were carried out in 12-week-old C57BL/6 mice. Mice not injected with B16-F10 cells (NT) were used as controls. Mice were also treated with the S1PR1 functional antagonist FTY720 and/or the S1PR5 functional antagonist BAF312, starting 1 week before the tumor cell injection. (**A**) Tumor growth as assessed by luminescence. (**B**) Bone perforations and ectopic bone growth as assessed by μCT. (**C**) μCT indices of cortical structure measured in tibial diaphysis. *n =* 5 mice per group. Data are expressed as mean ± SEM. All data were normally distributed and were analyzed by 2-way ANOVA and post hoc tests applying Bonferroni’s correction for multiple comparisons. *P < 0.05, **P < 0.01, ***P < 0.001, ****P < 0.0001 compared with the indicated group. Nonsignificant comparisons are not shown.

In mice subjected to intracardiac injection of B16-F10 cells, treatment with FTY720 or BAF312 alone or in combination similarly increased tumor growth at days 10 and 13 (Figure 7A), worsened tumor-induced metaphyseal perforations and ectopic bone growth (Figure 7B), worsened midshaft cortical bone loss without affecting bone area (Figure 7C), and aggravated metaphyseal trabecular bone loss (Supplemental Figure 12A). Comparable effects of treatment with FTY720 and/or BAF312 were found in mice subjected to intratibial injection of B16-F10 cells. In fact, in this experimental model, treatment with FTY720 or BAF312 or with both increased tumor growth at 5, 10, and 15 days (Supplemental Figure 13A), worsened cortical perforation indices and ectopic bone growth (Supplemental Figure 13B), aggravated cortical bone loss without affecting bone area (Supplemental Figure 13C), and worsened trabecular bone loss (Supplemental Figure 12B). No significant differences were detected between these effects of FTY720, BAF312, or combined FTY720 and BAF312 treatment.

### CXCR3 and CXCL9 induce the influx of Th1 cells and NK1 cells into the BM.

**Figure 8:**
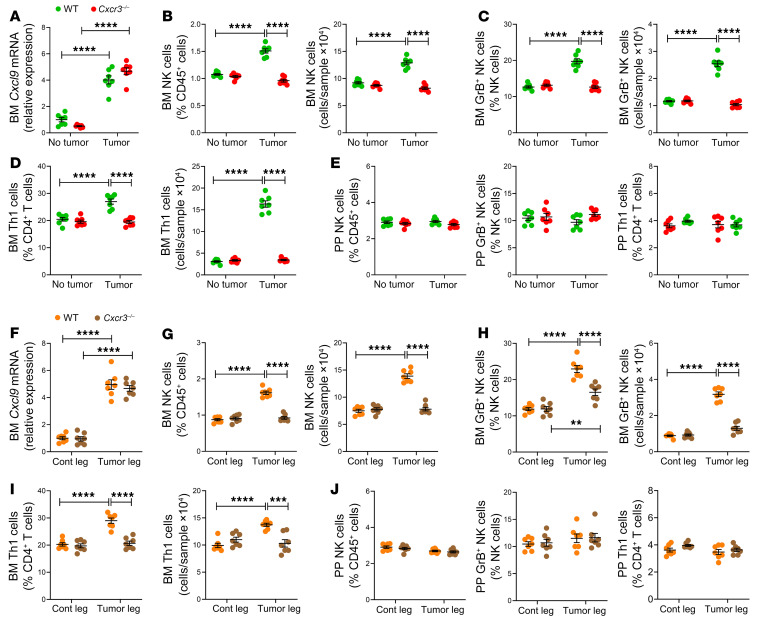
Global deletion of CXCR3 prevents the expansion of BM NK and Th1 cells induced by intracardiac and intratibial injection of melanoma cells. Intracardiac (**A**–**E**) and intratibial injections (**F**–**J**) of B16-F10 melanoma cells were carried out in 12-week-old C57BL/6 WT and *Cxcr3^–/–^* mice. In the intracardiac model, mice not injected with B16-F10 cells (No tumor) were used as controls. In the intratibial model, the noninjected contralateral leg (Cont leg) was used as control. (**A** and **F**) BM levels of CXCL9 transcripts. (**B** and **G**) Relative and absolute frequency of BM NK cells. (**C** and **H**) Relative and absolute frequency of BM GrB^+^ NK cells. (**D** and **I**) Relative and absolute frequency of BM Th1 cells. (**E** and **J**) Relative frequency of PP NK cells, GrB^+^ NK cells, and Th1 cells. *n =* 7 mice per group. Data are expressed as mean ± SEM. All data were normally distributed and were analyzed by 2-way ANOVA and post hoc tests applying Bonferroni’s correction for multiple comparisons. **P < 0.01, ***P < 0.001, ****P < 0.0001 compared with the indicated group. Nonsignificant comparisons are not shown.

**Figure 9:**
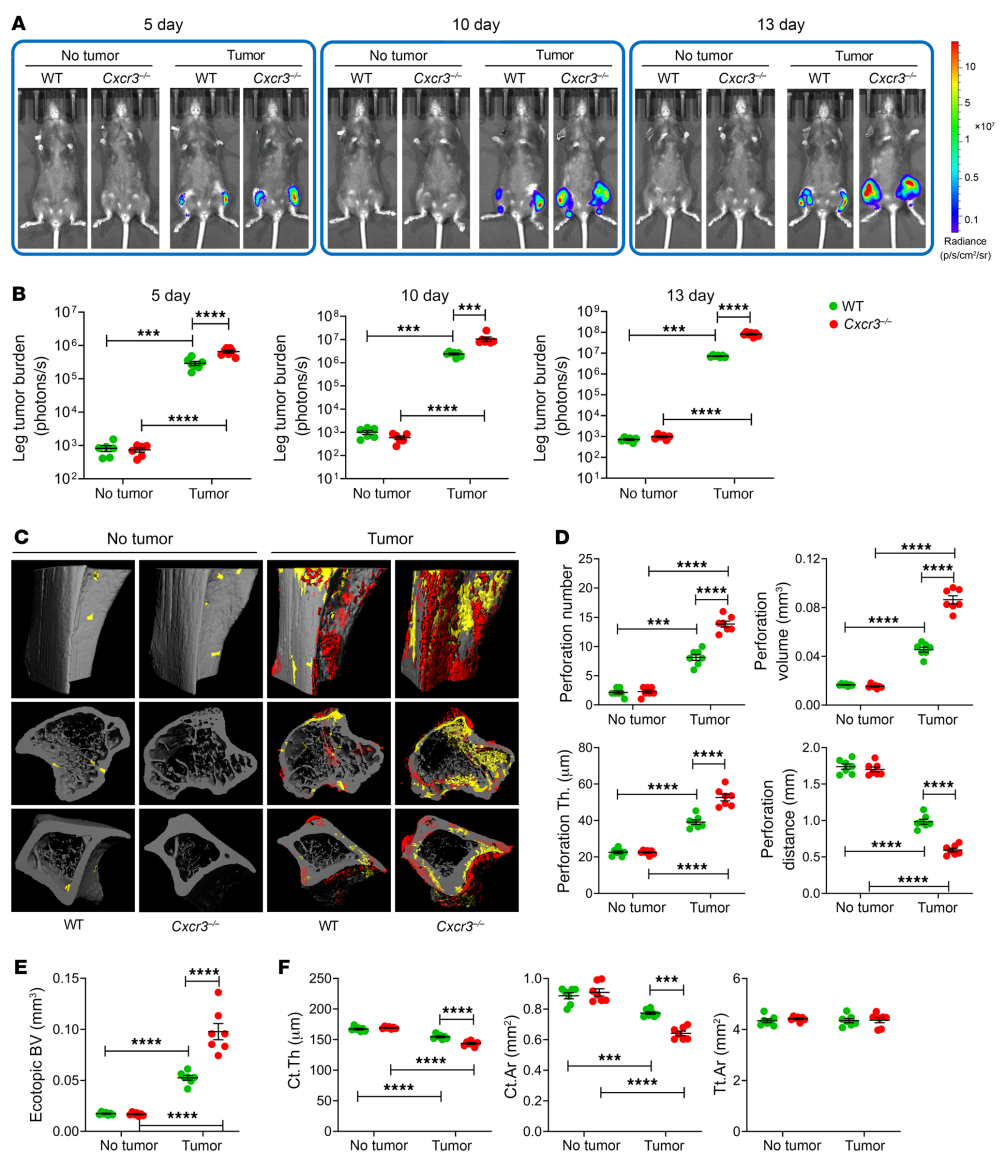
Silencing of CXCR3 accelerates bone tumor growth induced by intracardiac injection of melanoma cells. Mice not injected with B16-F10 cells (No tumor) were used as controls. (**A** and **B**) Tumor growth at day 13 as assessed by luminescence. (**C**–**E**) Bone perforations and ectopic bone growth as assessed by μCT. (**C**) Representative images of the tibia. Red pseudocolor, perforations; yellow pseudocolor, ectopic bone growth. (**D** and **E**) Indices of perforation and ectopic bone formation. (**F**) μCT indices of cortical structure measured in tibial diaphysis. *n =* 7 mice per group. Data are expressed as mean ± SEM. All data were normally distributed and were analyzed by 2-way ANOVA and post hoc tests applying Bonferroni’s correction for multiple comparisons. ***P < 0.001, ****P < 0.0001 compared with the indicated group. Nonsignificant comparisons are not shown.

**Figure 10:**
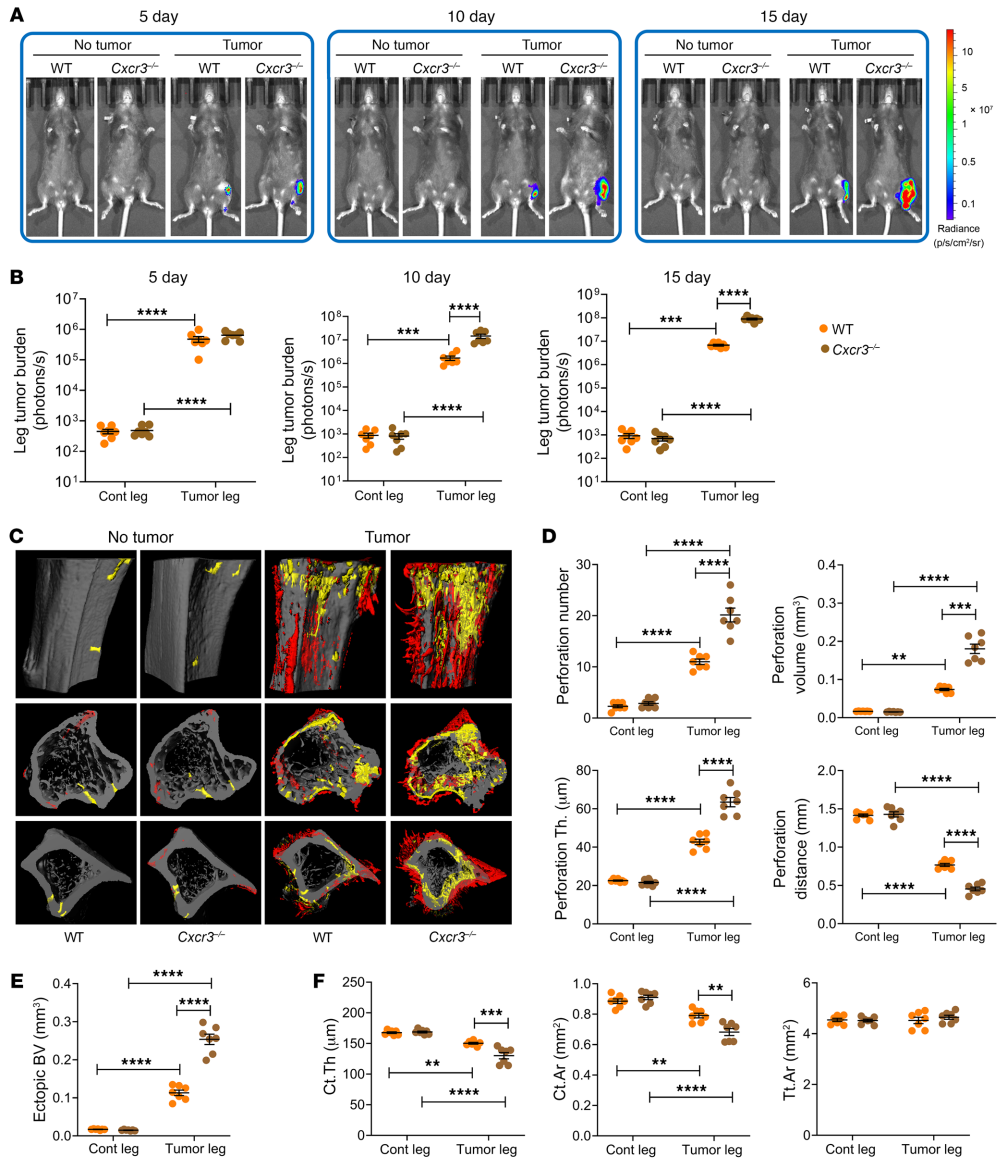
Silencing of CXCR3 accelerates bone tumor growth induced by intratibial injection of melanoma cells. The noninjected contralateral leg (Cont leg) was used as control. (**A** and **B**) Tumor growth at day 15 as assessed by luminescence. (**C**–**E**) Bone perforations and ectopic bone growth as assessed by μCT. (**C**) Representative images of the tibia. Red pseudocolor, perforations; yellow pseudocolor, ectopic bone growth. (**D** and **E**) Indices of perforation and ectopic bone formation. (**F**) μCT indices of cortical structure measured in tibial diaphysis. *n =* 7 mice per group. Data are expressed as mean ± SEM. All data were normally distributed and were analyzed by 2-way ANOVA and post hoc tests applying Bonferroni’s correction for multiple comparisons. **P < 0.01, ***P < 0.001, ****P < 0.0001 compared with the indicated group. Nonsignificant comparisons are not shown.

While the egress of Th1 and NK cells from intestinal lymphoid tissues and their entrance into the bloodstream are driven by the S1PR1/5 receptors and their ligand S1P, the exit from the systemic circulation of Th1 and NK cells and their infiltration of cancers are driven by CXCR3, a receptor expressed by T cells (44, 45) and NK cells (46), and its ligand CXCL9, which is induced or upregulated by IFN-γ (47, 48). To determine the relevance of CXCR3 to melanoma-dependent NK and Th1 cell migration to the BM, intracardiac or intratibial injections of B16-F10 cells were performed in 12-week-old *Cxcr3^–/–^* mice and WT littermate controls. In both the intracardiac and the intratibial injection model, BM levels of *Cxcl9* transcripts increased in response to B16-F10 cell growth in both WT and *Cxcr3^–/–^* mice (Figure 8, A and F). By contrast, the frequency of BM NK cells, GrB^+^ NK cells, and Th1 cells was increased by bone tumor growth in WT but not in *Cxcr3^–/–^* mice (Figure 8, B–D and G–I). As a result, the frequency of NK and Th1 T cells in the BM of B16-F10 cell–injected mice was lower in *Cxcr3^–/–^* mice than in WT mice, underscoring the critical role of CXCR3 in NK and Th1 T cell homing to the BM. As expected, the frequency of these cells in PPs was similar in WT and *Cxcr3^–/–^* mice (Figure 8, E and J). Attesting to the functional relevance of these effects, measurement of tumor growth by luminescence revealed that *Cxcr3^–/–^* mice had increased tumor growth as compared with WT mice (Figure 9, A and B, and Figure 10, A and B). In addition, *Cxcr3^–/–^* mice had more severe cortical perforation indices (Figure 9, C and D, and Figure 10, C and D), ectopic bone growth (Figure 9E and Figure 10E), cortical bone loss (Figure 9F and Figure 10F), and trabecular bone loss (Supplemental Figure 14, A and B). Together, these data provided evidence that CXCR3 functions in inducing the influx of Th1 cells and NK1 cells into the BM and impacts bone tumor growth.

**Figure 11:**
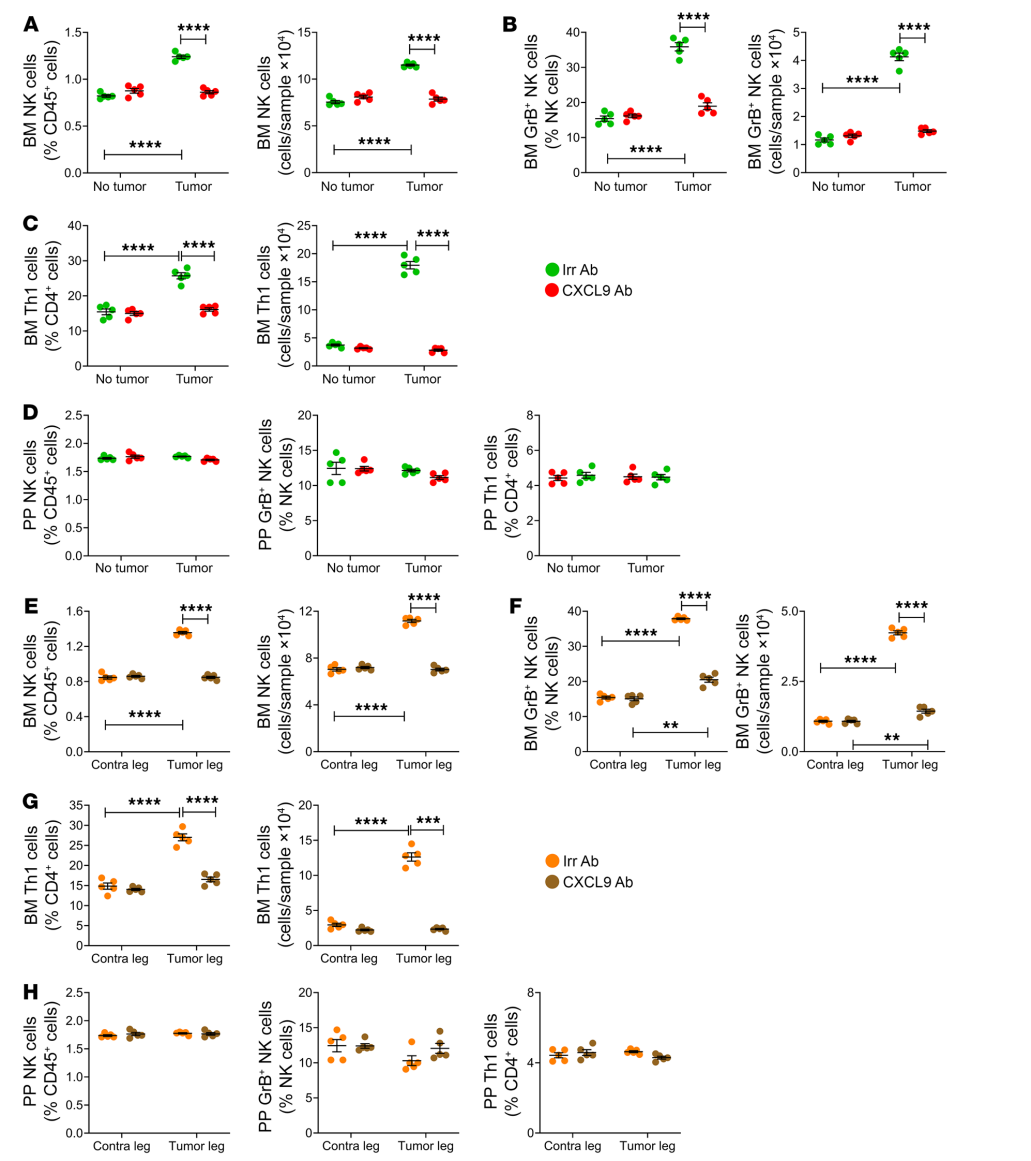
Antibody neutralization of CXCL9 prevents the expansion of BM NK and Th1 cells induced by intracardiac and intratibial injection of melanoma cells. Intracardiac (**A**–**D**) and intratibial injections (**E**–**H**) of B16-F10 melanoma cells were carried out in 12-week-old mice. In the intracardiac model, mice not injected with B16-F10 cells (No tumor) were used as controls. In the intratibial model, the noninjected contralateral leg (Cont leg) was used as control. Mice were treated with anti-CXCL9 antibody or isotype-matched irrelevant antibody. (**A** and **E**) Relative and absolute frequency of BM NK cells. (**B** and **F**) Relative and absolute frequency of BM GrB^+^ NK cells. (**C** and **G**) Relative and absolute frequency of BM Th1 cells. (**D** and **H**) Relative frequency of PP NK cells, GrB^+^ NK cells, and Th1 cells. *n =* 5 mice per group. Data are expressed as mean ± SEM. All data were normally distributed and were analyzed by 2-way ANOVA and post hoc tests applying Bonferroni’s correction for multiple comparisons. **P < 0.01, ***P < 0.001, ****P < 0.0001 compared with the indicated group. Nonsignificant comparisons are not shown.

**Figure 12:**
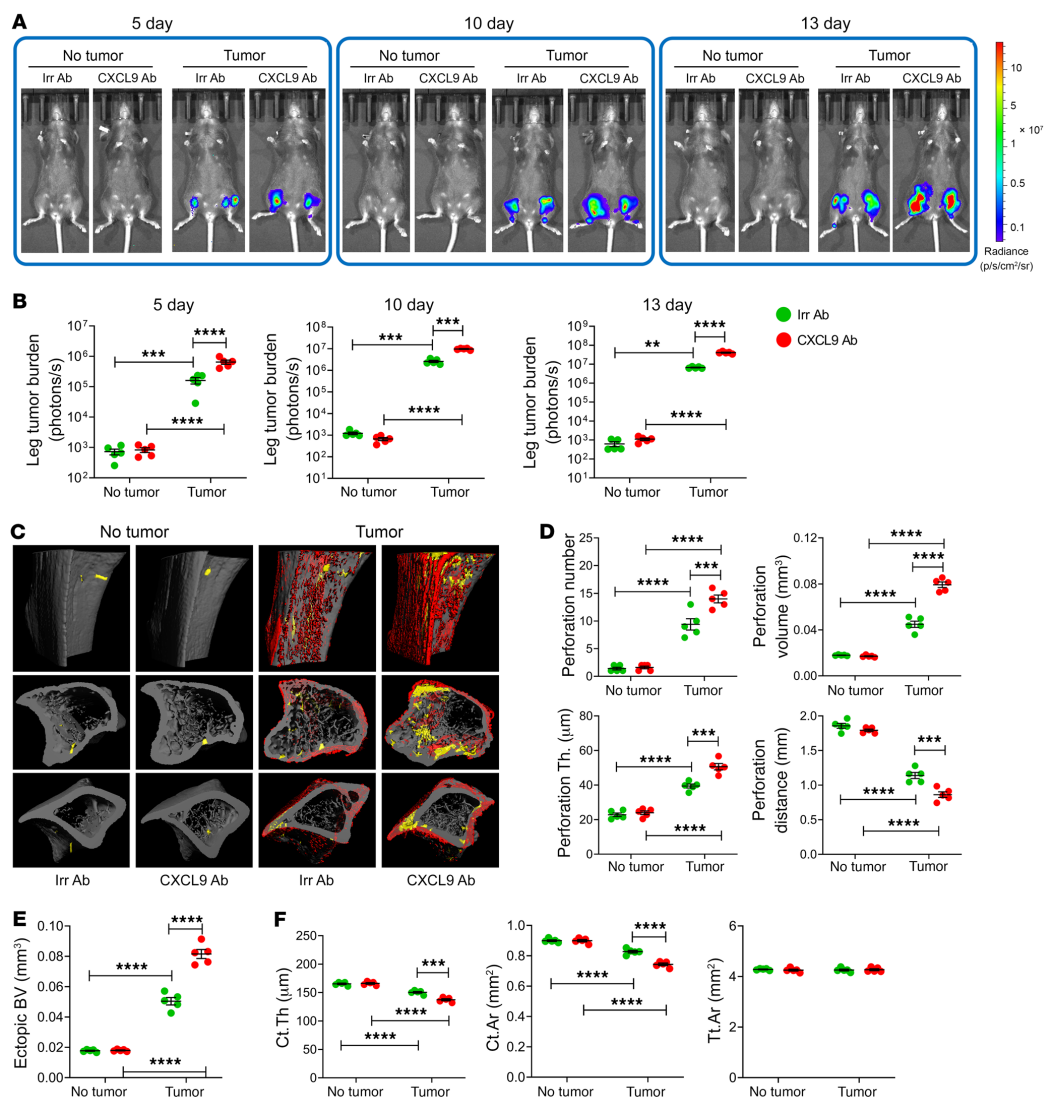
Antibody neutralization of CXCL9 accelerates bone tumor growth induced by intracardiac injection of melanoma cells. Mice not injected with B16-F10 cells (No tumor) were used as controls. (**A** and **B**) Tumor growth at day 13 as assessed by luminescence. (**C**–**E**) Bone perforations and ectopic bone growth as assessed by μCT. (**C**) Representative images of the tibia. Red pseudocolor, perforations; yellow pseudocolor, ectopic bone growth. (**D** and **E**) Indices of perforation and ectopic bone formation. (**F**) μCT indices of cortical structure measured in tibial diaphysis. *n =* 5 mice per group. Data are expressed as mean ± SEM. All data were normally distributed and were analyzed by 2-way ANOVA and post hoc tests applying Bonferroni’s correction for multiple comparisons. **P < 0.01, ***P < 0.001, ****P < 0.0001 compared with the indicated group. Nonsignificant comparisons are not shown.

**Figure 13:**
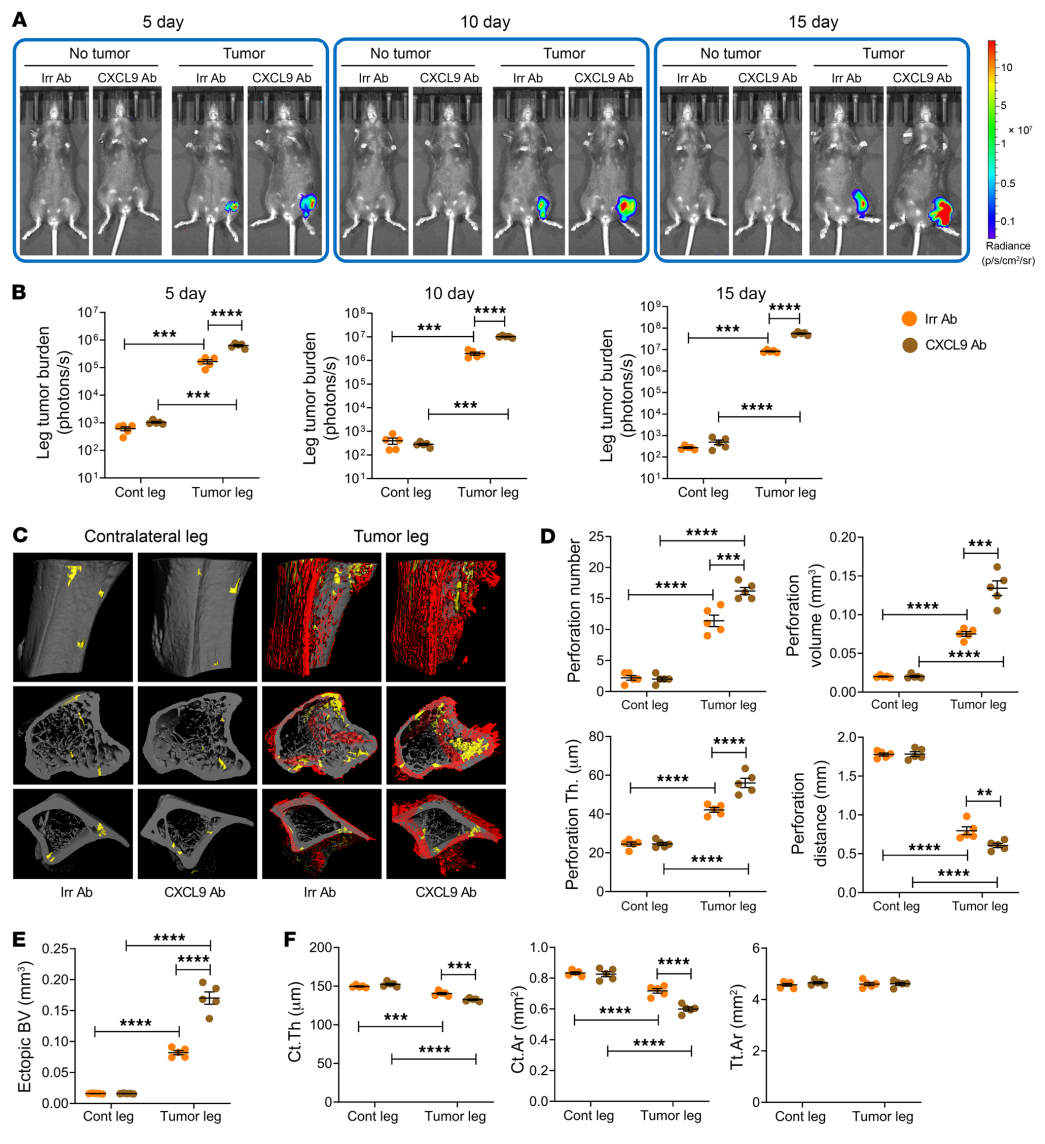
Antibody neutralization of CXCL9 accelerates bone tumor growth induced by intratibial injection of melanoma cells. The noninjected contralateral leg (Cont leg) was used as control. (**A** and **B**) Tumor growth at day 15 as assessed by luminescence. (**C**–**E**) Bone perforations and ectopic bone growth as assessed by μCT. (**C**) Representative images of the tibia. Red pseudocolor, perforations; yellow pseudocolor, ectopic bone growth. (**D** and **E**) Indices of perforation and ectopic bone formation. (**F**) μCT indices of cortical structure measured in tibial diaphysis. *n =* 5 mice per group. Data are expressed as mean ± SEM. All data were normally distributed and were analyzed by 2-way ANOVA and post hoc tests applying Bonferroni’s correction for multiple comparisons. **P < 0.01, ***P < 0.001, ****P < 0.0001 compared with the indicated group. Nonsignificant comparisons are not shown.

To investigate the role of the CXCR3 ligand CXCL9, intracardiac or intratibial injections of B16-F10 cells were carried out in 12-week-old WT mice. Mice were treated with anti-CXCL9 antibody or isotype-matched irrelevant antibody. In both the intracardiac and the intratibial injection model, the frequency of BM NK cells, GrB^+^ NK cells, and Th1 cells was increased by bone tumor growth in control mice but not in those treated with anti-CXCL9 antibody (Figure 11, A–C and E–G). By contrast, the frequency of NK cells, GrB^+^ NK cells, and Th1 cells in PPs was similar in all groups (Figure 11, D and H). Attesting to the functional relevance of CXCL9, measurement of tumor growth by luminescence revealed that mice treated with anti-CXCL9 antibody had increased tumor growth as compared with those treated with irrelevant antibody (Figure 12, A and B, and Figure 13, A and B). In addition, CXCL9 neutralization led to more severe cortical perforations (Figure 12, C and D, and Figure 13, C and D), ectopic bone growth (Figure 12E and Figure 13E), cortical bone loss (Figure 12F and Figure 13F), and trabecular bone loss (Supplemental Figure 15, A and B).

## Discussion

We report that the gut microbiome restrains the progression of melanoma bone lesions in mice by promoting the expansion of intestinal NK cells and Th1 cells and enhancing their migration to the BM of tumor-bearing bones. To directly investigate the effect of tumor growth in bone on immune cell trafficking, we made use of Kaede mice (35). This strain has been successfully used to track the migration of intestinal immune cells to the BM (8), kidney (49), mesenteric lymph nodes (50), and brain (51). These experiments revealed that expansion of melanoma cells in bone increases the tropism of NK and Th1 cells for the BM in a microbiome-dependent fashion. The homing of NK and Th1 cells to the BM was specifically directed to the BM within the cancer lesions, as these cells were not attracted to cancer-free spleen or liver. We did not find evidence that bone cells or bone-produced factors are required for intestinal NK and Th1 cells to home to the BM of tumor-bearing bones, but a possible contribution of bone-specific factors remains to be conclusively determined. These findings provide novel mechanistic insights into the role of the microbiome in the development of bone lesions in melanoma, an aggressive cancer that frequently affects the skeleton.

The egress of NK and Th1 cells from the gut and their entrance into the bloodstream are driven by the S1P receptor S1PR5 expressed by NK cells (39, 46), the S1PR1 receptor expressed by Th1 cells (40, 41), and the S1PR1/5 circulating ligand S1P (39). In this study the relevance of intestinal NK cells in regulating the abundance of NK cells in the BM was demonstrated using BAF312, an S1PR5 modulator that arrests NK cell exit from the BM and intestinal lymphoid tissues (42, 43). We found that BAF312 did not affect the number of intestinal NK cells. By contrast, even though BAF312 blunted NK cells’ exit from the BM, the frequency of NK cells in the BM within cancer lesions was markedly decreased by BAF312 in control and B16-F10 cell–injected mice. Moreover, BAF312 prevented the tumor-induced increase in BM NK cells, indicating that an influx of intestinal NK cells into the BM contributes to the tumor-induced increase in the frequency of BM NK cells.

The role of intestinal Th1 cells in regulating the size of the pool of BM Th1 cells was revealed by FTY720, an S1PR1 modulator that arrests the exit of all lymphocytes from the intestine to the systemic circulation (40, 41). This agent lowered the frequency of BM Th1 cells without affecting the frequency of intestinal Th1 cells. Moreover, FTY720 prevented the tumor-induced increase in BM Th1 cells, demonstrating the relevance of intestinal Th1 cells in regulating the frequency of BM Th1 cells. Since FTY270 and BAF312 block the intestinal egress of all lymphocytes (42, 43), it could be argued that increased cancer growth induced by these agents might have resulted, in part, from the impaired migration of lymphocytes capable of restraining cancer growth other than NK and Th1 cells. This hypothesis is unlikely because the acceleration of bone cancer growth induced by FTY270 and/or BAF312 was comparable to the effects of antibiotics, which blocked the migration of NK cells and Th1 cells only. We found the influx of NK and Th1 cells to the BM within cancer lesions to be dependent on the chemokine receptor CXCR3, which is expressed on NK cells (46) and T cells (44, 45), and on its ligand CXCL9, which is induced by IFN-γ (47, 48, 52). Accordingly, the BM cell expression of CXCL9 was upregulated by B16-F10 cell expansion, while silencing of either the CXCR3 receptor or its ligand CXCL9 blunted the increase in the frequency of BM NK cells and Th1 cells induced by B16-F10 cell expansion.

Attesting to the relevance of intestinal NK cells and Th1 cells, the S1P-S1PR1/5 axes, and the CXCR3-CXCL9 chemokine gradient in restraining bone tumor growth, blockade of NK and Th1 cell egress from the intestine or blockade of their influx into the BM resulted in accelerated cancer growth in bone, an effect mirroring the consequences of microbiome ablation by antibiotics. Simultaneous blockade of NK cells or Th1 cell trafficking potentiated cancer growth as effectively as migration inhibition of one cell lineage only, a finding consistent with the existence of reciprocal control of the anticancer activity of these 2 lineages (53). Since FTY720 is currently FDA approved for the treatment of multiple sclerosis (MS), the potential negative effect of FTY720 in cancer burden may need to be considered if this drug is used in patients affected by melanoma and MS.

NK cells play a key role in the immune response to melanoma (53) and other cancers (21). Upon forming immune synapses with target cells, NK cells induce cell lysis by releasing cytolytic granules, including perforin and granzymes (54). In addition to their direct anticancer activity, NK cells promote Th1 polarization through multiple mechanisms (53, 55), including IFN-γ production (56). Th1 cells are primarily responsible for activating tumor antigen–specific cytotoxic T lymphocytes (CTLs) (22). This activation is mediated by direct cell contact (57), through cytokines (58), or via the involvement of intermediary cell lineages. In addition, Th1 cells kill tumor cells via release of cytokines that activate death receptors on the tumor cell surface. A common effector molecule of NK cells and Th1 cells is IFN-γ. This explains, in part, the overlapping anticancer activities of these 2 lineages. IFN-γ enhances tumor immunogenicity through the upregulation of MHC class I expression on tumor cells, thereby making tumor cells sensitive to CTL-mediated elimination (59). IFN-γ also has antiproliferative, antiangiogenic, and proapoptotic effects against tumor cells (60–62). IFN-γ is also a critical player in the migration of NK cells to tumor sites. In fact, IFN-γ induces the expression of the CXCR3 ligand CXCL9 (47), driving the influx of NK cells to the tumor bed.

Antibiotics-induced dysbiosis was previously reported by other investigators to accelerate cancer growth (63–65). Jenkins et al. reported that injection of B16-F10 melanoma cells resulted in more rapid local cancer growth in antibiotic-treated mice. Antibiotics were found to decrease the expression of TNF-α, which resulted in a decrease in the number of effector T cells (63). In another study, preexisting dysbiosis caused primary mammary tumors to expand more rapidly in the breast. Dysbiosis also increased tumor cell dissemination to the lungs by enhancing inflammation and fibrosis in the mammary gland (64). Recently, McKee et al. reported that antibiotics resulted in accelerated breast tumor growth via an increase in the number of mast cells in tumor stroma (65). However, the current study is, to our knowledge, the first to provide evidence for a role of the gut microbiome in the progression of bone metastasis and provide information about trafficking of immune cells from the gut to BM within bone lesions.

The role of the microbiota in regulating the contributions of other cell lineages and cytokines implicated in the immune response to melanoma remains to be determined, as our investigation was mostly focused on NK and Th1 cells, lineages known to be directly regulated by the gut microbiota, for which biochemical reagents are available to follow their migration from the gut to the BM. Microbial metabolites are also known to regulate the immune response to cancer. Butyrate was one of the first bacterial products implicated in cancer progression and response to immune therapy (66). Novel metabolites and mechanisms of action have been recently described. For example, it has been reported that microbiota-derived stimulators of interferon gene agonists induce production of IFN-1, a factor promoting NK cell–dendritic cell crosstalk, by intratumor monocytes, thus improving the efficacy of immune checkpoint blockade in melanoma patients (32). Additional studies will be required to elucidate the multiple mechanisms by which bacteria and their products affect cancer progression and response to therapy, either directly or via regulatory activities on intestinal and tumor-microenvironment immune cells.

In summary, this study reveals mechanisms for microbiota-mediated gut-bone crosstalk in mouse models of metastatic melanoma relevant to the immunological restraint of bone cancer growth. The observation that microbiome depletion accelerates metastatic bone growth indicates that antibiotics and other causes of dysbiosis may have unforeseen adverse consequences in melanoma patients.

## Methods

### Mice.

C57BL/6NTac mice were purchased from Taconic Biosciences. *Cxcr3^–/–^* mice (B6.129P2-Cxcr3<tm1Dgen>/J) were purchased from The Jackson Laboratory. C57BL/6 Kaede mice [B6.Cg-c/c Tg(CAG-tdKaede)15Utr] were purchased from RIKEN BioResource Research Center. All mice entering Emory University were shipped to the same room in the same vivarium. All mice were housed under specific pathogen–free conditions and were fed γ-irradiated 5V5R mouse chow (Purina Mills) and autoclaved water ad libitum. The animal facility was kept at 23°C (± 1°C) with 50% relative humidity and a 12-hour light/12-hour dark cycle. All mice were acclimatized within our facility for at least 3 days before experimentation.

### Intracardiac and intratibial B16-F10 cell injection.

Luciferase-expressing B16-F10 melanoma cells (1 × 10^5^) in 100 μL of PBS were injected into the left cardiac ventricle or the tibia of 12-week-old female mice as previously described (67–68). The mice were monitored daily for 13–15 days and then sacrificed. For intracardiac injection experiments, control mice without tumors were generated by injection of PBS only. Control samples for intratibial injection experiments were generated by injection of PBS in the contralateral tibia. PPs from intracardiac-injection no-tumor groups were used as control PPs for the intratibial injection experiments.

### Depletion of gut commensal microflora.

Cocktails of broad-spectrum antibiotics (1 mg/mL ampicillin, 0.5 mg/mL vancomycin, 1 mg/mL neomycin sulfate, and 1 mg/mL metronidazole; ref. 70) or of nonabsorbable antibiotics (2 mg/mL bacitracin and 2 mg/mL neomycin sulfate) were included in the drinking water starting at 2 weeks before initiation of cancer cell injection and continued until sacrifice. Fecal microbiome depletion was verified by fecal DNA extraction using QIAamp DNA Stool Mini Kit (QIAGEN) and subsequent quantitative PCR using an established protocol that used primers specific for the detection of the 16S rRNA gene present in all bacteria (5′-GTGCCAGCMGCCGCGGTAA-3′, forward; 5′-GGACTACHVGGGTWTCTAAT-3′, reverse), as previously described (9, 10).

### FTY720, BAF312, and anti-CXCL9 antibody treatment.

The S1PR1 functional antagonist FTY720 (49) was added to the drinking water at 5 μg/mL as previously described (8, 9). The S1PR5 functional antagonist BAF312 was delivered by oral gavage (3 mg/kg) as previously described (71). FTY720 and BAF312 treatments were initiated 1 week before B16-F10 cell injection and continued until sacrifice. Drinking water containing FTY720 was changed weekly. Anti–mouse CXCL9 antibody (catalog BE0309, Bio X Cell) or isotype-matched irrelevant antibody (catalog BE0091, Bio X Cell) was injected i.p. every other day at 100 μg per mouse, starting 3 days before tumor cell injection and continued until sacrifice, as previously described (72, 73).

### In vivo imaging system–based analysis of tumor growth.

Mice were injected s.c. with 150 mg/kg d-luciferin potassium salt (P/N 122799, PerkinElmer), anesthetized using isoflurane, and imaged 15 minutes after injection as previously described (68, 74). Imaging was performed on days 5, 10, and 13 for the intracardiac injection model and on days 5, 10, and 15 for the intratibial injection model. For analysis, total photon flux (photons per second) was measured from a fixed region of interest (ROI) in the tumor area using Living Image 4.50 software.

### μCT-based analysis of tumor-induced cortical perforations and ectopic bone formation at the femoral and tibial metaphysis.

Excised hind limbs were fixed in 10% neutral-buffered formalin, then imaged in a μCT40 (Scanco Medical AG). Images were acquired in the axial plane at 70 peak kilovoltage, 114 μA, 500 projections per rotation, and an integration time of 300 milliseconds, then reconstructed using the 1200 mg HA/cm^3^ beam hardening correction to generate axial images with 12.0 μm isotropic voxels. Extraperiosteal orthotopic new bone as well as cortical perforations and connected surface erosions in the proximal tibial and distal femoral metaphysis was analyzed using a semiautomated algorithm. Briefly, analysis began with drawing of a “loose” volume of interest (VOI) outside the metaphyseal cortex leaving at least 100 μm between the contour and the bone surface, whether cortical or heterotopic, on the axial images at 360 μm to 2400 μm from the proximal tibial or distal femoral growth plate. The method of Buie et al. (75) was modified to create masks approximating the smooth periosteal and endosteal surfaces that span small cortical perforations and erosions, rather than dipping into the surface imperfections. A 3D reconstruction of medullary marrow space and cortical imperfections that extend to the periosteal surface was then created by application of a lower threshold of 0 and an upper threshold of 450 mg HA/cm^3^, sigma 0.8, and support 2 to the volume inside the periosteal contour. Extraperiosteal space and cortical perforations that extended to the endosteal surface were segmented in a similar manner by combination of the hand-drawn “loose” outer contour with the automated endosteal contour and application of the same inverse threshold. The intramedullary and extraperiosteal volumes were concatenated, and the cortical perforations and surface erosions connected to the perforations were identified as the overlapping volumes present in both objects. The endocortical and periosteal contours were combined to create a cortical mask of the smoothed bone surfaces inclusive of perforations and erosions, which served to define total cortical volume for calculation of volumetric and architectural parameters of the perforations. Standard procedures that are commonly applied to the analysis of trabecular bone (76–78) were then applied to the perforation images to calculate cortical volume, perforation volume, perforation number, and perforation thickness.

Ectopic new bone extending from the periosteal surface was segmented by combination of the periosteal and hand-drawn “loose” contours to create the extraperiosteal VOI, then application of a threshold of 180 mg HA/cm^3^ without the Gaussian noise filter. The volumetric and architectural parameters of ectopic bone were calculated using standard procedures and nomenclature (76–78).

### μCT-based analysis of metaphyseal trabecular and midshaft cortical bone volume and architecture.

Trabecular and cortical volume and architecture in the proximal tibial metaphysis, distal femoral metaphysis, and femoral midshaft were analyzed by μCT as previously reported (79–81) and using the same acquisition settings as above. The VOI for trabecular analysis was the entire medullary volume lying 0.24 mm to 0.84 mm from the central tip of the growth plate, and trabecular bone was segmented from soft tissue using a threshold of 425 mg HA/cm^3^. Midshaft cortical bone was analyzed in 50 axial μCT slices comprising the middle 0.60 mm of the femur. The periosteal and endosteal masks were created as previously described (75), and cortical bone was segmented using a threshold of 700 mg HA/cm^3^, sigma 0.2, and support 1. Cortical and trabecular volumetric and architectural parameters were calculated using standard methods (76–78).

### PP and BM single-cell suspension.

For PP cell isolation, the small intestine (SI) was removed and flushed of fecal content. PPs were excised and collected in 1 mL cooled RPMI 1640. PPs were dissociated using the plunger of a 2.5 mL syringe and gently forced through a 70 μm cell strainer placed over a 50 mL tube. A single-cell suspension was used for flow cytometric analysis. For BM cell isolation, tumor-bearing femur and tibia were flushed with PBS, and BM cells were collected. RBC lysis was performed twice to eliminate all the RBCs from BM. Single-cell suspension of BM cells was used for analysis by flow cytometry, as previously described (9, 10).

### Kaede mouse cell photoconversion.

Kaede mice express a photoconvertible fluorescence protein that changes from green (518 nm) to red (582 nm) upon exposure to near-UV (350–410 nm) light. Twelve-week-old segmented filamentous bacteria–positive (SFB^+^) Kaede mice were subjected to intracardiac or intratibial B16-F10 tumor cell injection. Nine days later, animals underwent laparotomy, during which the cecum and distal SI were eviscerated and the 4 PPs most proximal to the cecum were identified and illuminated with 390 nm wavelength light for 2 minutes each. Aluminum foil was used to protect tissue other than target PPs from light during exposure. Mice were sacrificed 24 or 48 hours after photoconversion, and PP cells were collected. A single-cell suspension was prepared for analysis by flow cytometry. BM cells were also collected at sacrifice and a single-cell suspension prepared. BM KaedeR NK and Th1 cells were enumerated by flow cytometry by analysis of whole BM cells.

### Flow cytometry.

Flow cytometry was performed on a FACS Cytek Aurora (Cytek Biosciences), and data were analyzed using FlowJo software (Tree Star Inc.). Antibodies and reagents used for surface and intracellular staining are listed in Supplemental Table 1. The live cells were discriminated by LIVE/DEAD Fixable Yellow Dead Cell Stain Kit (Thermo Fisher Scientific). For intracellular staining, cells were incubated with cell activation cocktail (BioLegend) containing PMA, ionomycin, and monensin at 37°C for 12 hours. Antibodies were added after cell fixation and permeabilization.

### Real-time reverse transcriptase PCR and primers.

Total RNA was isolated using TRIzol reagent (Thermo Fisher Scientific) according to the manufacturer’s protocol. For all RNA samples, cDNA was synthesized from mRNA using the SuperScript III First Strand kit (Invitrogen). The expression levels of CXCL9 were measured in tumor-bearing BM cells by real-time PCR. Changes in relative gene expression between groups were calculated by the 2^–ΔΔCt^ method with normalization by 18S rRNA. Primer sequences used for the experiment were as follows: CXCL9, 5′-AACGTTGTCCACCTCCCTTC-3′ (forward) and 5′-CACAGGCTTTGGCTAGTCGT-3′ (reverse); 18S, 5′-ATTCGAACGTCTGCCCTATCA-3′ (forward) and 5′-GTCACCCGTGGTCACCATG-3′ (reverse).

### Statistics.

All data are expressed as mean ± SEM. All data were normally distributed according to the Shapiro-Wilk normality test. Data were analyzed by 2-way ANOVA. This analysis included the main effects for tumor versus no tumor and treatment plus the statistical interaction between tumor versus no tumor and treatment. When the statistical interaction was statistically significant (*P <* 0.05) or suggestive of an important interaction, then unpaired 2-tailed *t* tests were used to compare the differences between the treatment means for each animal strain, applying Bonferroni’s correction for multiple comparisons.

### Study approval.

All animal procedures were approved by the Institutional Animal Care and Use Committee of Emory University in compliance with all applicable federal regulations governing the protection of animals in research.

## Author contributions

SP, MNW, RF, and RP designed the studies. SP, DSP, TY, JA, and AS performed the research and analyzed the animal data. SP, DSP, RF, CMC, RMJ, MNW, and RP wrote the manuscript.

## Supplementary Material

Supplemental data

## Version 1 05/03/2022

In-Press Preview

## Version 2 06/15/2022

Electronic publication
