# Supplementary material for: The microbiome restrains melanoma bone growth by promoting intestinal NK and Th1 cell homing to bone
Source: J Clin Invest. 2022 Jun 15;132(12):e157340. doi: 10.1172/JCI157340 (PMC9197523; doi:10.1172/JCI157340)
Supplement: Supplemental data [file jci-132-157340-s191.pdf]

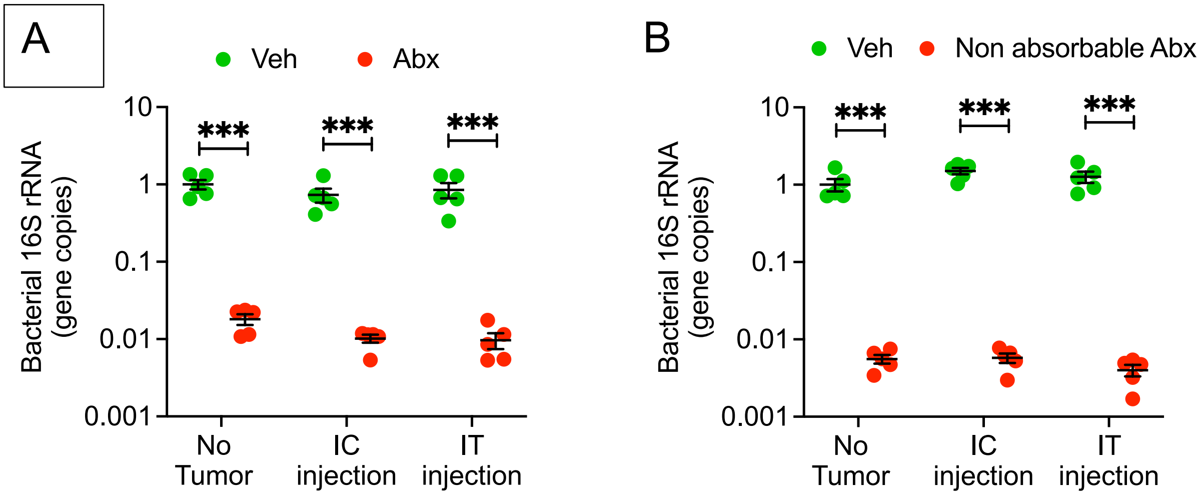

SF 1. Analysis of fecal bacterial-specific DNA. (A) Effect of broad-spectrum antibiotics (Abx). **(B)** Effect of non-absorbable antibiotics. Intracardiac (IC) or intratibial (IT) injections of the luciferase expressing B16-F10 melanoma cell line were carried out in 12-week-old female C57BL/6 mice. Mice were treated with either broad-spectrum antibiotics (1 mg/mL ampicillin, 0.5 mg/mL vancomycin, 1 mg/mL neomycin sulfate, 1 mg/mL metronidazole administered in drinking water) or non-absorbable antibiotics (1 mg/mL neomycin sulfate, 1 mg/mL bacitracin dissolved in drinking water) for 4 weeks, starting 2 weeks before the tumor cell injection. Genomic DNA was isolated from equal amounts of fecal material. Relative quantitation of 16S rRNA gene copies of total bacteria were determined by qPCR, using universal 16S rRNA primers. Abx = antibiotics. n = 5 mice per group. Data are expressed as Mean ± SEM. Two-way analysis-of-variance and post hoc tests applying the Bonferroni correction for multiple comparisons. ***=p<0.001 compared to the indicated group.

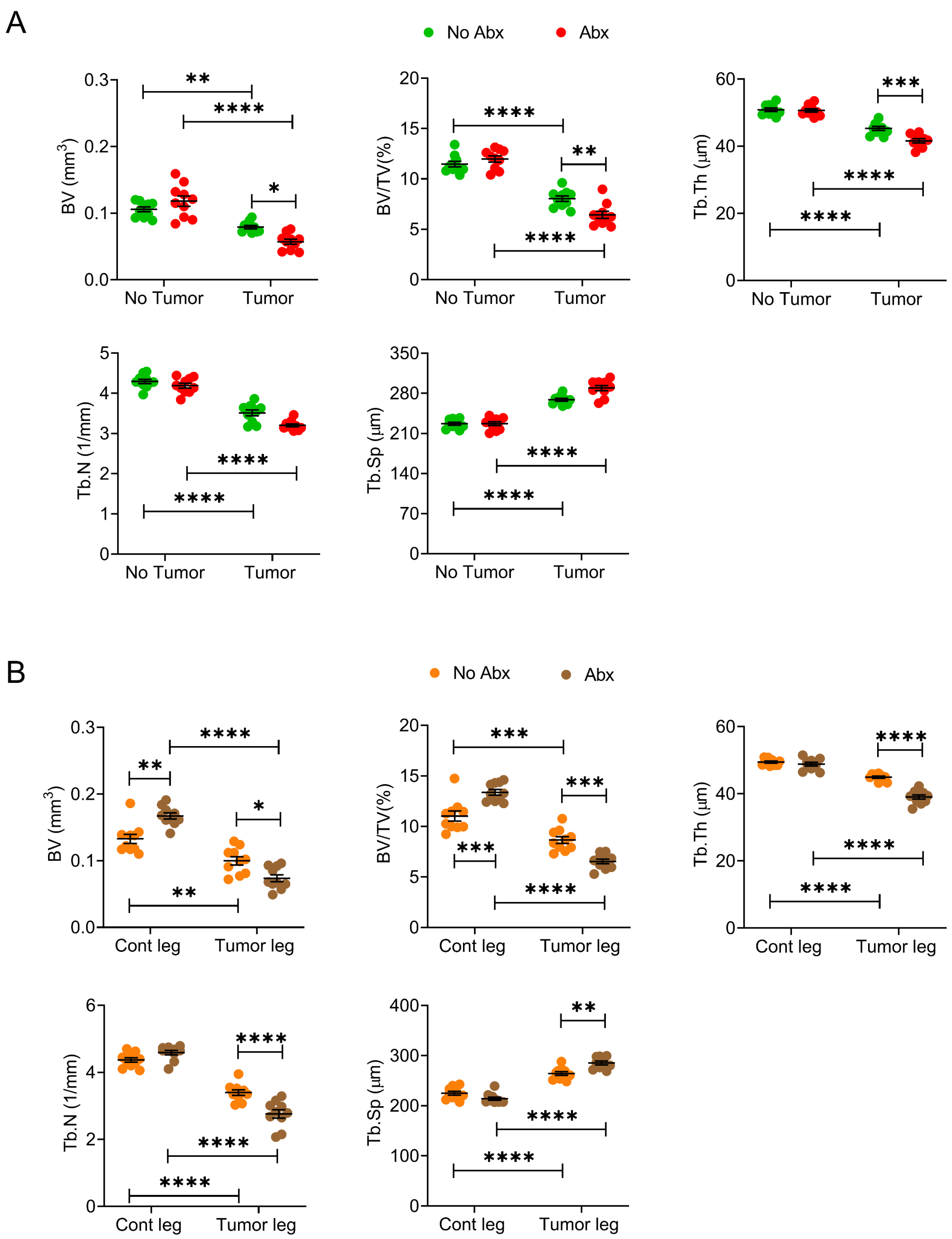

SF 2. Antibiotics-induced microbiota depletion worsens indices of trabecular structure caused by intracardiac and intratibial injections of melanoma cells. Intracardiac (Panel A) and intratibial (Panel B) injections of B16-F10 melanoma cell line were carried out in 12-week-old female C57BL/6 mice. In the intracardiac model, mice not injected with B16-F10 cells (No tumor) were used as controls. In the intratibial model, the non-injected contralateral leg (Cont leg) was used as control. Mice were treated with broad-spectrum antibiotics (1 mg/mL ampicillin, 0.5 mg/mL vancomycin, 1 mg/mL neomycin sulfate, 1 mg/mL metronidazole dissolved in water) for 4 weeks, starting 2 weeks before the tumor cell injection. n= 10 mice per group. Data are expressed as Mean ± SEM. All data were normally distributed according to the Shapiro-Wilk normality test and analyzed by two-way analysis-of-variance and post hoc tests applying the Bonferroni correction for multiple comparisons. *=p<0.05, **=p<0.01, ***=p<0.001 and ****= p<0.0001 compared to the indicated group. Nonsignificant comparisons not shown.

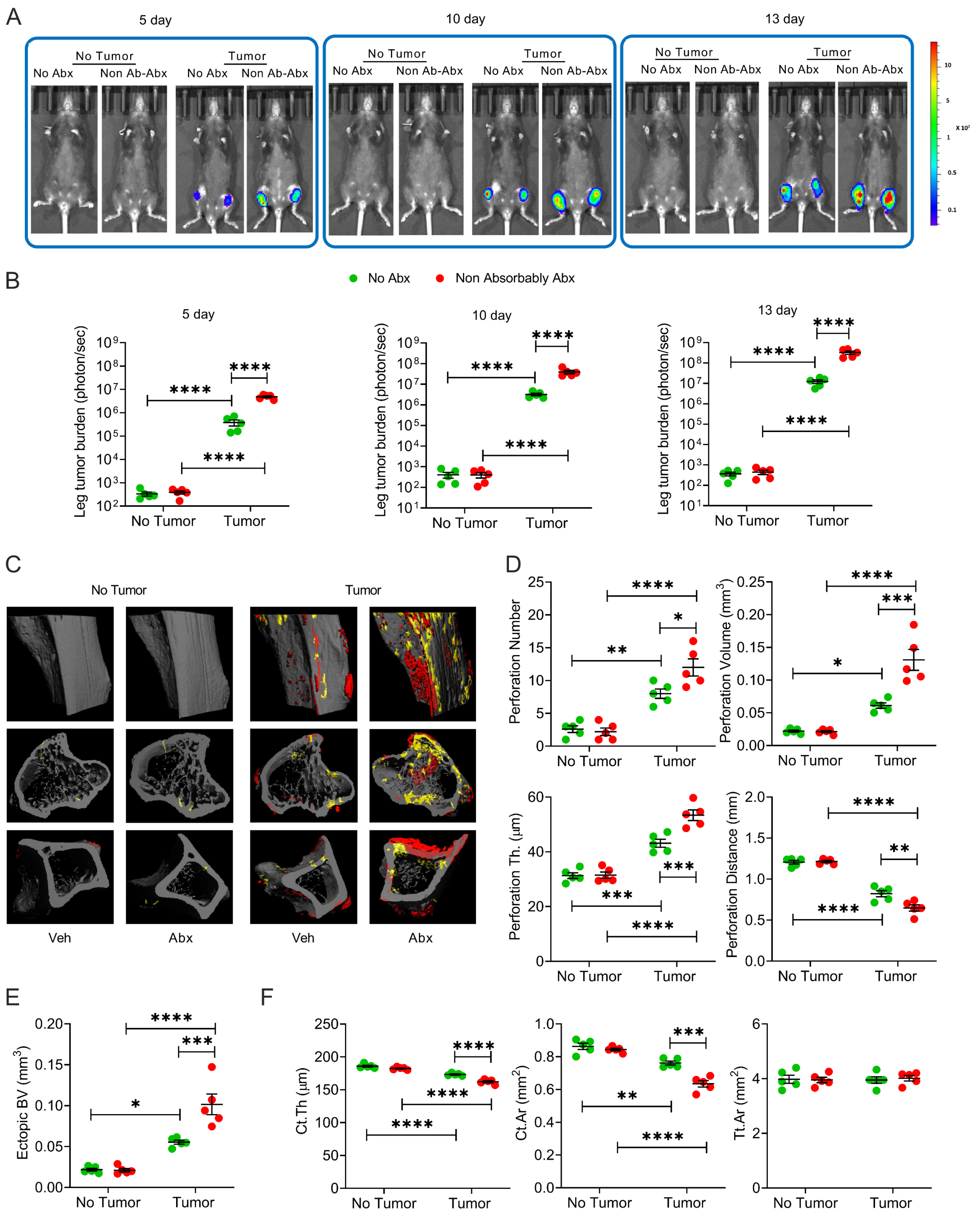

SF 3. Non-absorbable antibiotics-induced microbiota depletion accelerates bone tumor growth caused by intracardiac injections of melanoma cells. Intracardiac injections of luciferase expressing B16-F10 melanoma cell line were carried out in 12-week-old female C57BL/6 mice. Mice not injected with B16-F10 cells (No tumor) were used as controls. Mice were treated with non-absorbable antibiotics (2 mg/mL neomycin sulfate, and 2 mg/ml bacitracin delivered in drinking water) for 4 weeks, starting 2 weeks before the tumor cell injection. **(A,B)** Effects of antibiotics (Abx) an tumor growth as assessed by luminescence. (**C-E)** Effects of Abx on bone perforations and ectopic bone growth as assessed by µCT. Panel C shows representative images of the tibia. Red color = perforations, yellow color = ectopic bone growth. Panels D and E show indices of perforation and ectopic bone formation. (**F)** µCT indices of cortical structure measured in tibial diaphysis. n= 5 mice per group. Data are expressed as Mean ± SEM. All data were normally distributed according to the Shapiro-Wilk normality test and analyzed by two-way analysis-of-variance and post hoc tests applying the Bonferroni correction for multiple comparisons. *=p<0.05, **=p<0.01, ***=p<0.001 and ****= p<0.0001 compared to the indicated group. Nonsignificant comparisons not shown.

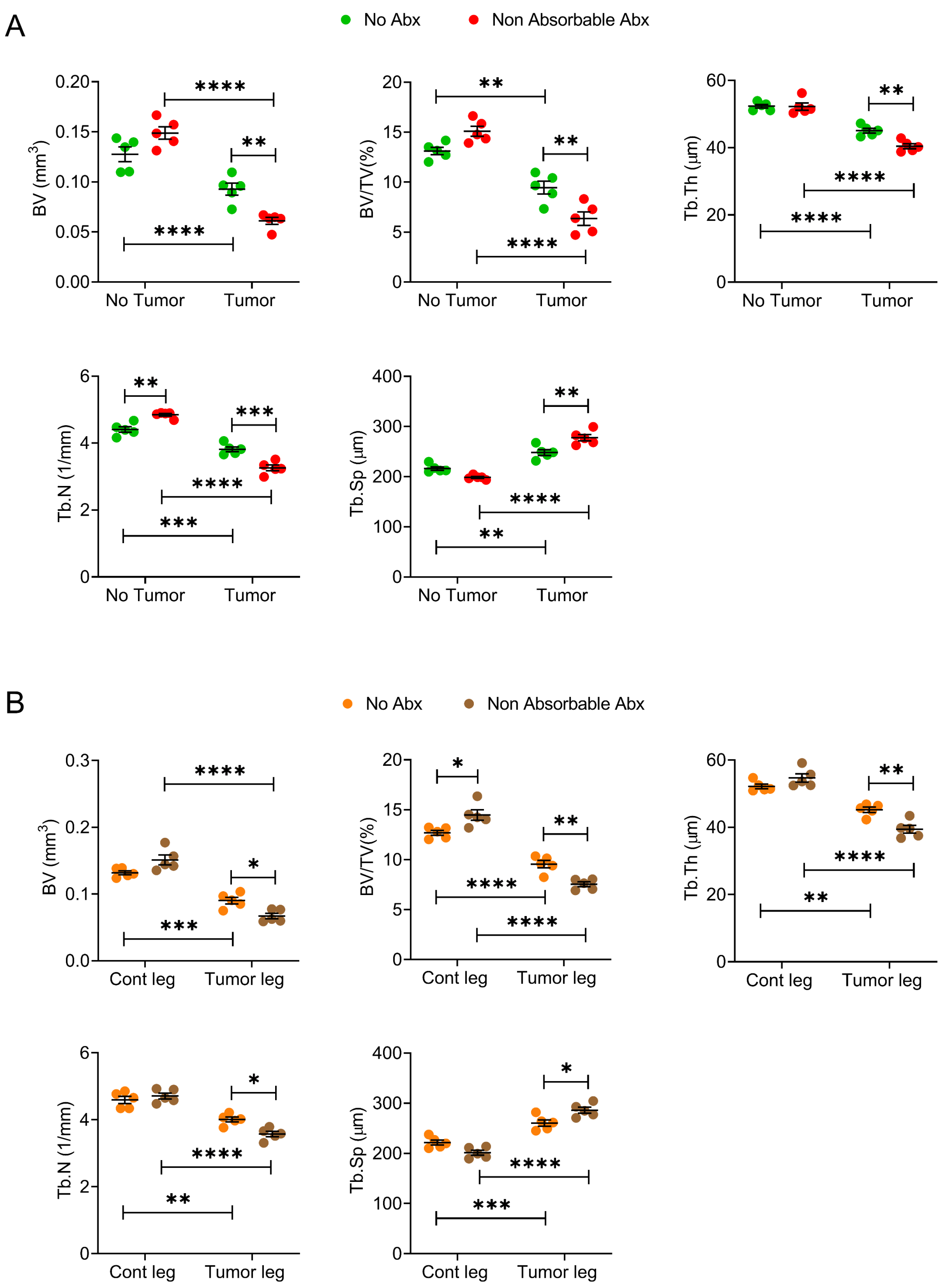

SF 4. Non-absorbable antibiotics worsens indices of trabecular structure caused by intracardiac and intratibial injections of melanoma cells. Intracardiac (Panel A) and intratibial (Panel B) injections of B16-F10 melanoma cell line were carried out in 12-week-old female C57BL/6 mice. In the intracardiac model, mice not injected with B16-F10 cells (No tumor) were used as controls. In the intratibial model, the non-injected contralateral leg (Cont leg) was used as control. Mice were treated with non-absorbable antibiotics (2 mg/mL neomycin sulfate, and 2 mg/ml bacitracin delivered in drinking water) for 4 weeks, starting 2 weeks before the tumor cell injection. n= 5 mice per group. Data are expressed as Mean ± SEM. All data were normally distributed according to the Shapiro-Wilk normality test and analyzed by two-way analysis-of-variance and post hoc tests applying the Bonferroni correction for multiple comparisons. *=p<0.05, **=p<0.01, ***=p<0.001 *and* ****= p<0.0001 compared to the indicated group. Nonsignificant comparisons not shown.

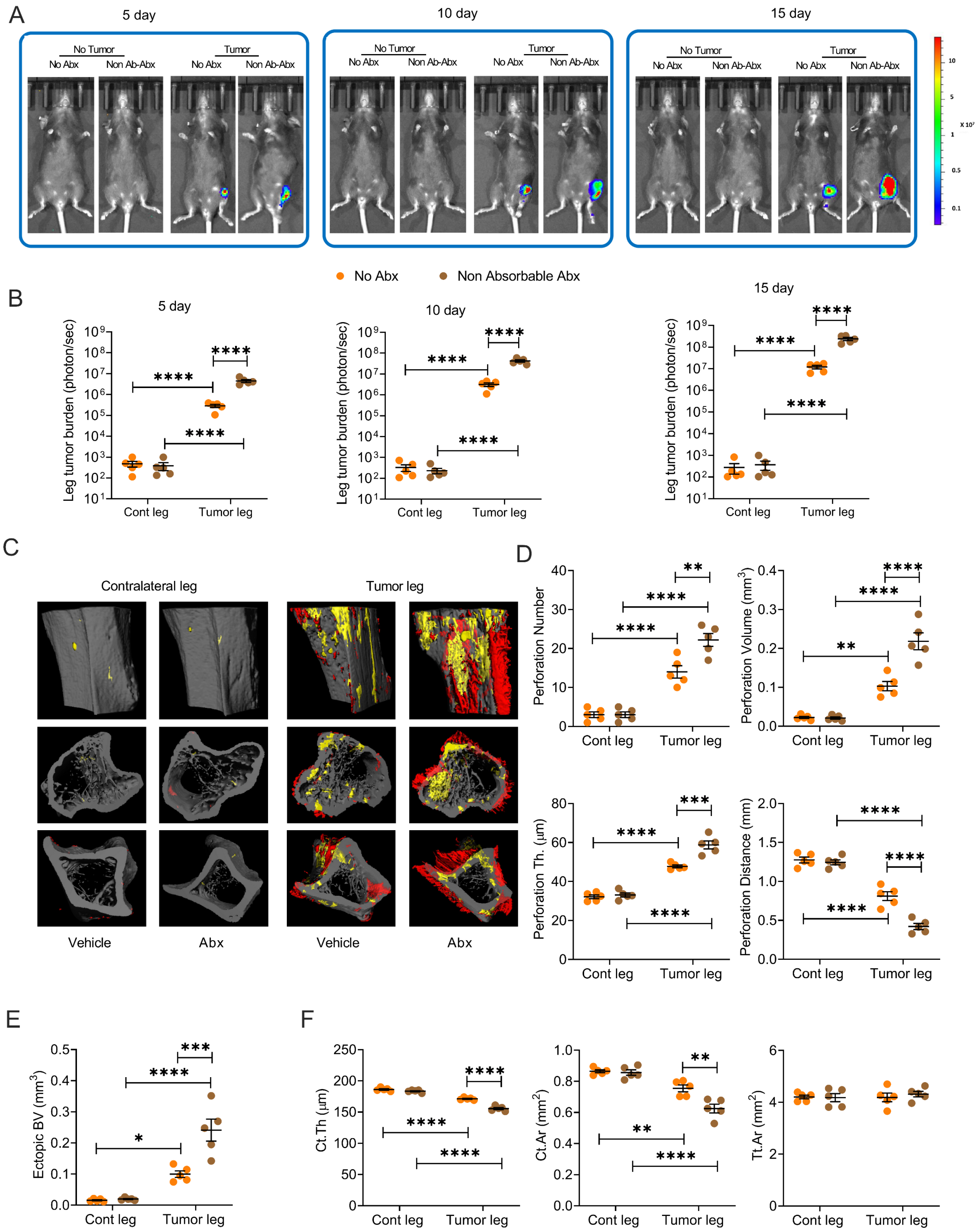

SF 5. Non-absorbable antibiotics-induced microbiota depletion accelerates bone tumor growth caused by intratibial injections of melanoma cells. Intracardiac injections of luciferase expressing B16-F10 melanoma cell line were carried out in 12-week-old female C57BL/6 mice. The non-injected contralateral leg (Cont leg) was used as a control. Mice were treated with non-absorbable antibiotics (2 mg/mL neomycin sulfate, and 2 mg/ml bacitracin delivered in drinking water) for 4 weeks, starting 2 weeks before the tumor cell injection. **(A,B)** Effects of antibiotics (Abx) an tumor growth as assessed by luminescence. **(C-E)**. Effects of Abx on bone perforations and ectopic bone growth as assessed by µCT. Panel C shows representative images of the tibia. Red color = perforations, yellow color = ectopic bone growth. Panels D and E show indices of perforation and ectopic bone formation. **(F)** µCT indices of cortical structure measured in tibial diaphysis. n= 5 mice per group. Data are expressed as Mean ± SEM. All data were normally distributed according to the Shapiro-Wilk normality test and analyzed by two-way analysis-of-variance and post hoc tests applying the Bonferroni correction for multiple comparisons. *=p<0.05, **=p<0.01, ***=p<0.001 and ****= p<0.0001 compared to the indicated group. Nonsignificant comparisons not shown.

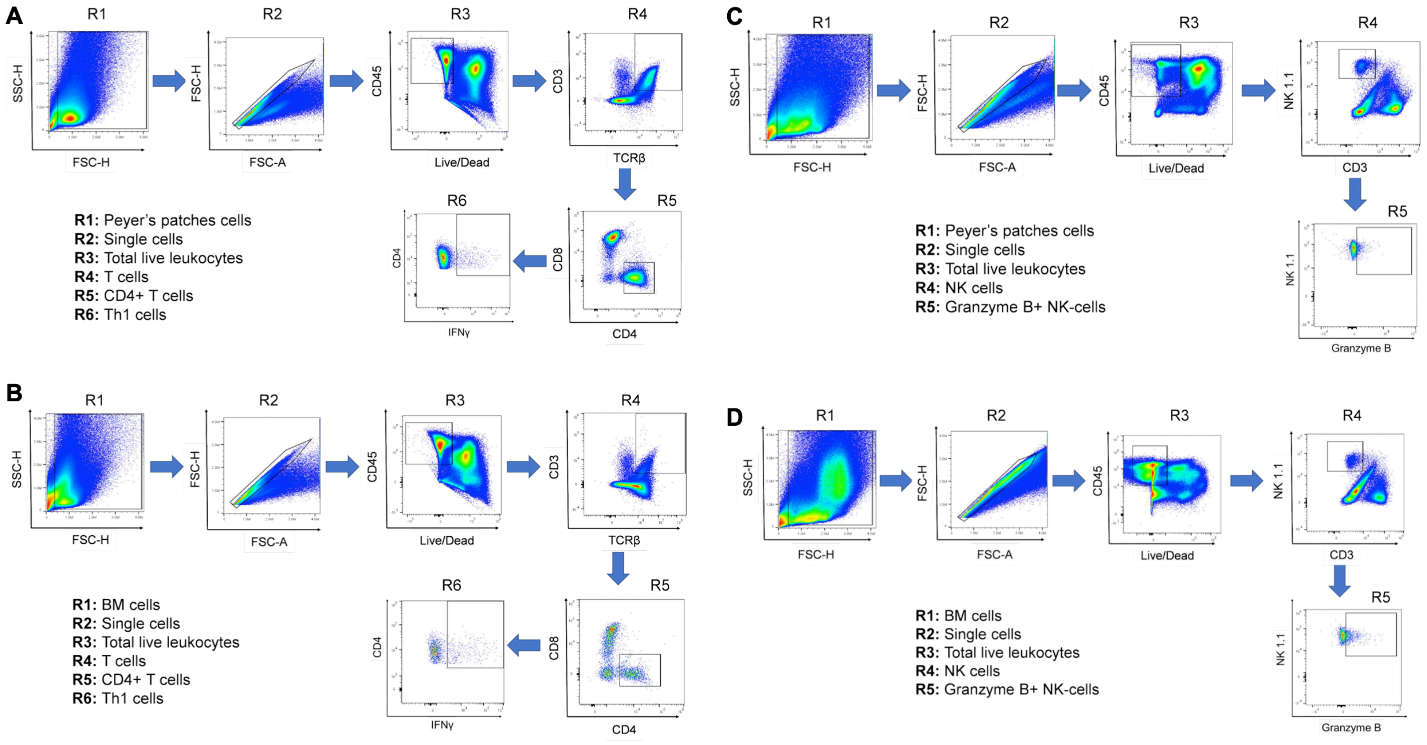

SF 6. **Gating strategy used to identify Th1 cells (Panels A,B) and NK cells (Panels C,D)**. Following red blood cells lysis, single cell suspensions were prepared from Payer's patches (panels A and C) and BM (panels B and D) and stained with antibodies to the indicated antigens and live/dead cell dye. Gated regions are numbered from R1 to R6. The figure shows one representative gating of flow cytometric plot.

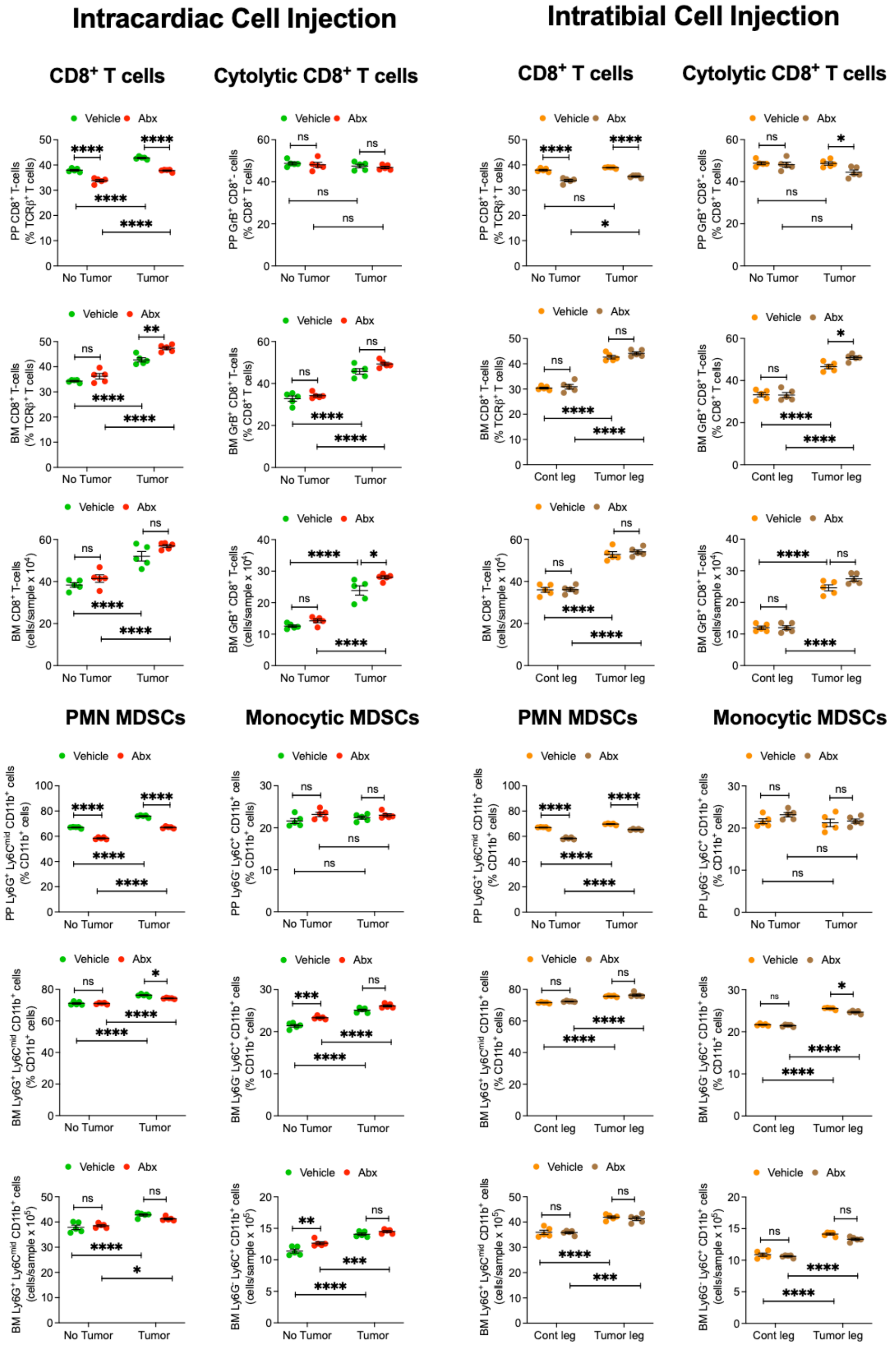

SF 7. Effects of antibiotics-induced microbiota depletion on PPs and BM CD8^{+} T cells, cytolytic CD8^{+} T cells polymorphonucleated (PMN) MDSCs and monocytic MDSCs in mice subjected to intracardiac and intratibial injections of melanoma cells. Injections of B16-F10 melanoma cells were carried out in 12-week-old female C57BL/6 mice. In the intracardiac model, mice not injected with B16-F10 cells (No tumor) were used as controls. In the intratibial model, the non-injected contralateral leg (Cont leg) was used as control. Mice were treated with broad-spectrum antibiotics (1 mg/mL ampicillin, 0.5 mg/mL vancomycin, 1 mg/mL neomycin sulfate, 1 mg/mL metronidazole dissolved in water) for 4 weeks, starting 2 weeks before the tumor cell injection. n= 5 mice per group. Data are expressed as Mean $\pm$ SEM. All data were normally distributed according to the Shapiro-Wilk normality test and analyzed by two-way analysis-of-variance and post hoc tests applying the Bonferroni correction for multiple comparisons. *=p<0.05, ***=p<0.001 and ****= p<0.0001 compared to the indicated group. Nonsignificant comparisons not shown.

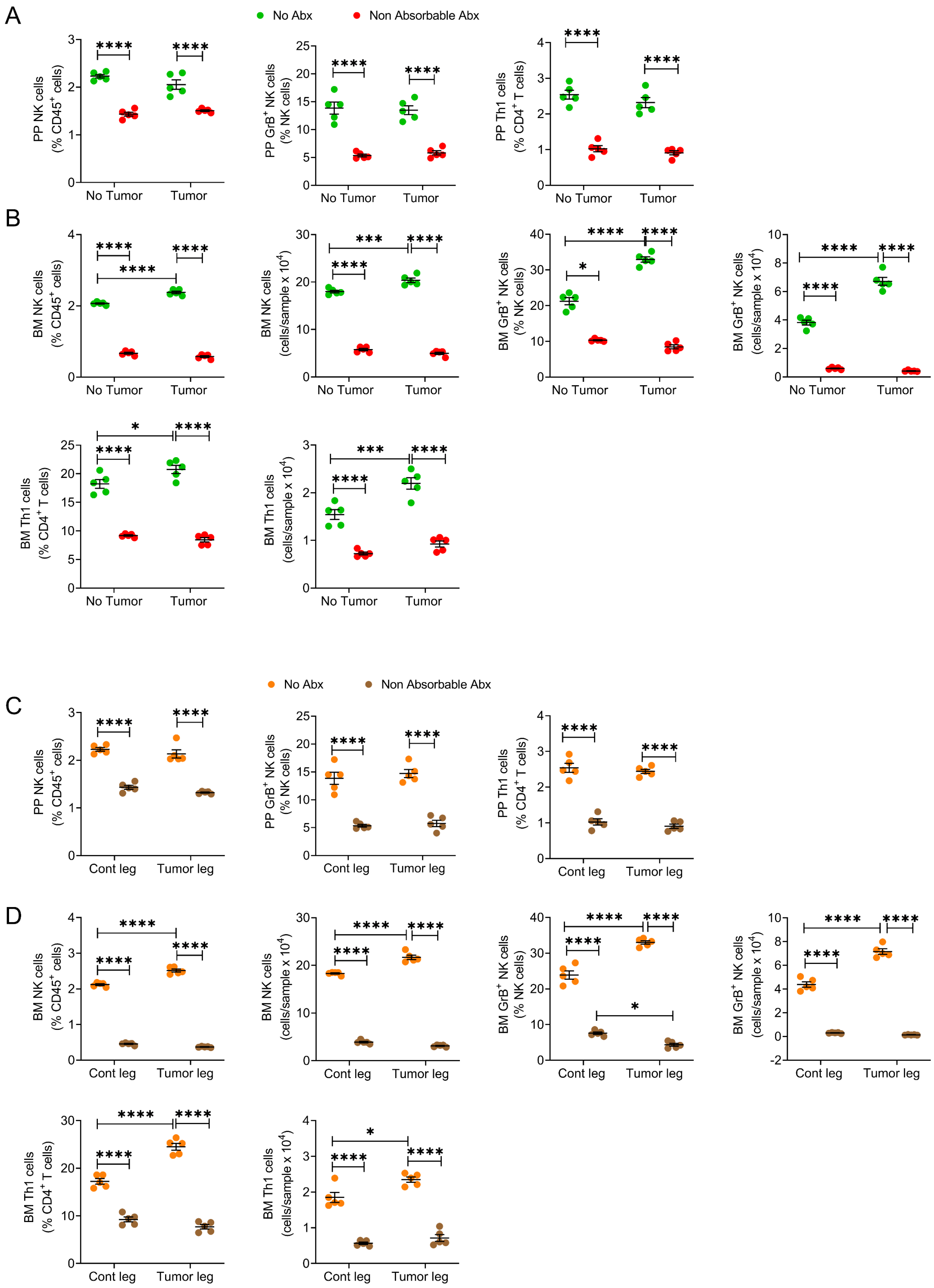

SF 8. Non-absorbable antibiotics-induced microbiota depletion prevents the expansion BM NK and Th1 cells induced by intracardiac and intratibial injections of melanoma cells. Intracardiac (panels A,B) and intratibial (panels C,D) injections of B16-F10 melanoma cells were carried out in 12-week-old female C57BL/6 mice. In the intracardiac model, mice not injected with B16-F10 cells (No tumor) were used as controls. In the intratibial model, the non-injected contralateral leg (Cont leg) was used as control. Mice were treated with non-absorbable antibiotics (2 mg/mL neomycin sulfate, and 2 mg/ml bacitracin delivered in drinking water) for 4 weeks, starting 2 weeks before the tumor cell injection. (**A,C**) Relative frequency of PP NK ($NK1.1^+CD3^+$) cells, Gr^+ NK cells and Th1 ($CD3^+CD4^+IFN\gamma^+$) cells. (**B,D**) Relative frequency of BM NK cells, Gr^+ NK cells and Th1 cells. n= 5 mice per group. Data are expressed as Mean ± SEM. All data were normally distributed according to the Shapiro-Wilk normality test and analyzed by two-way analysis-of-variance and post hoc tests applying the Bonferroni correction for multiple comparisons. *=p<0.05, ***=p<0.001 and ****= p<0.0001 compared to the indicated group. Nonsignificant comparisons not shown.

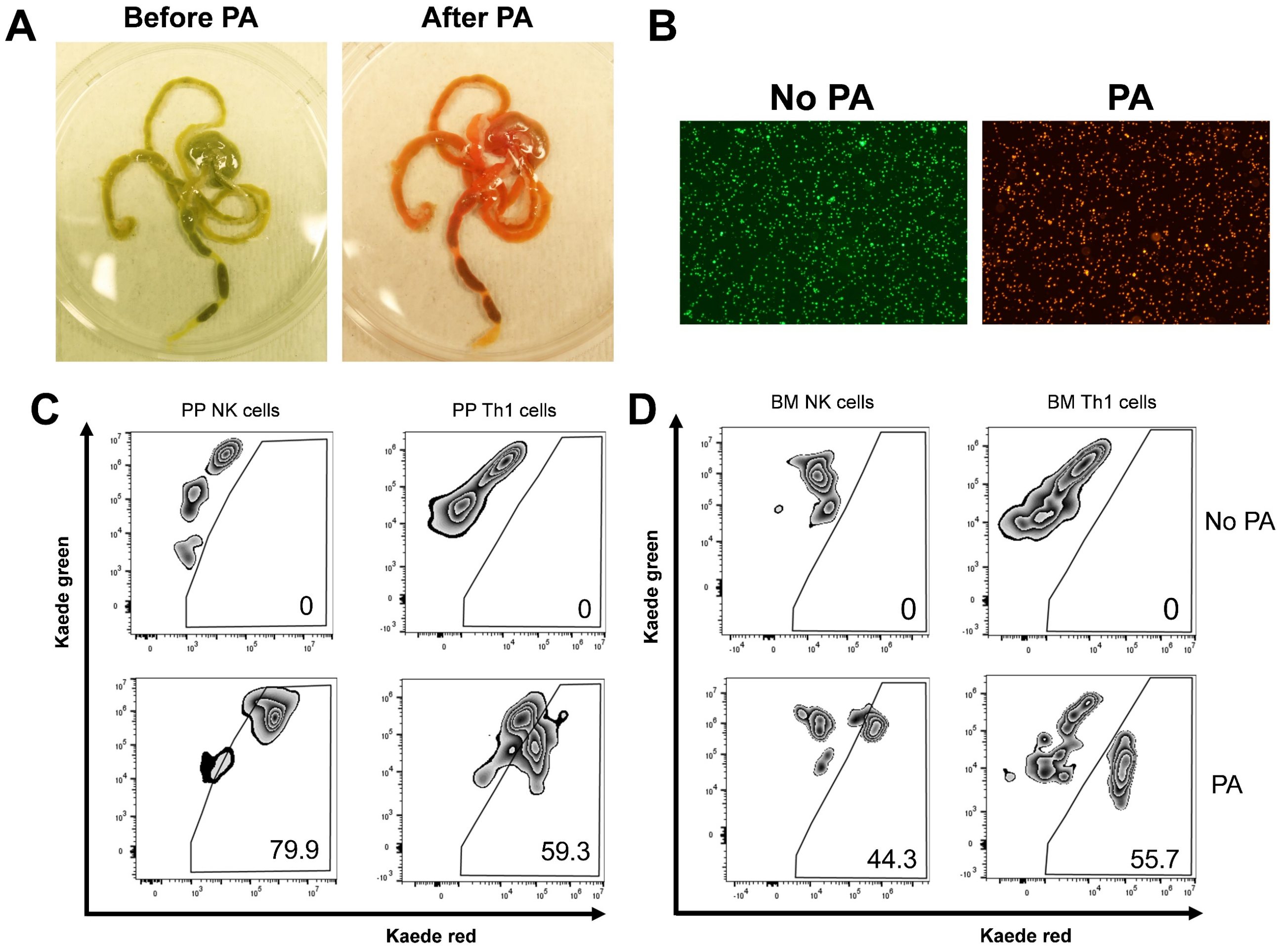

SF 9: Representative images and flow cytometric analysis of small intestines and Peyer's patch cells from Kaede mice subjected or not subjected to photoactivation (PA). (A) Images of the intestine of Kaede mice before and after ex vivo PA of the dissected organ by exposure to a 390 nm wavelength light for 2 minutes. (**B)** Image of Peyer's patch cells of Kaede mice subjected or not subjected to PA. PPs were harvested from intact mice and subjected or not subjected to ex vivo PA. PP cells were resuspended in PBS and imaged using a fluorescent microscope (10x magnification). (**C-D)** Representative flow cytometric plot of PP and BM NK cells and Th1 cells from one Kaede mouse subjected to intracardiac injection of B16-F10 cells. Laparotomy was performed 9 days later. PPs were photo-converted by exposing them to a 390 nm light for 2 minutes. The mouse was sacrificed 24hr after photoconversion. PP and BM cells were analyzed by flow. Control no PA samples were obtained at sacrifice from 1 intact Kaede mouse.

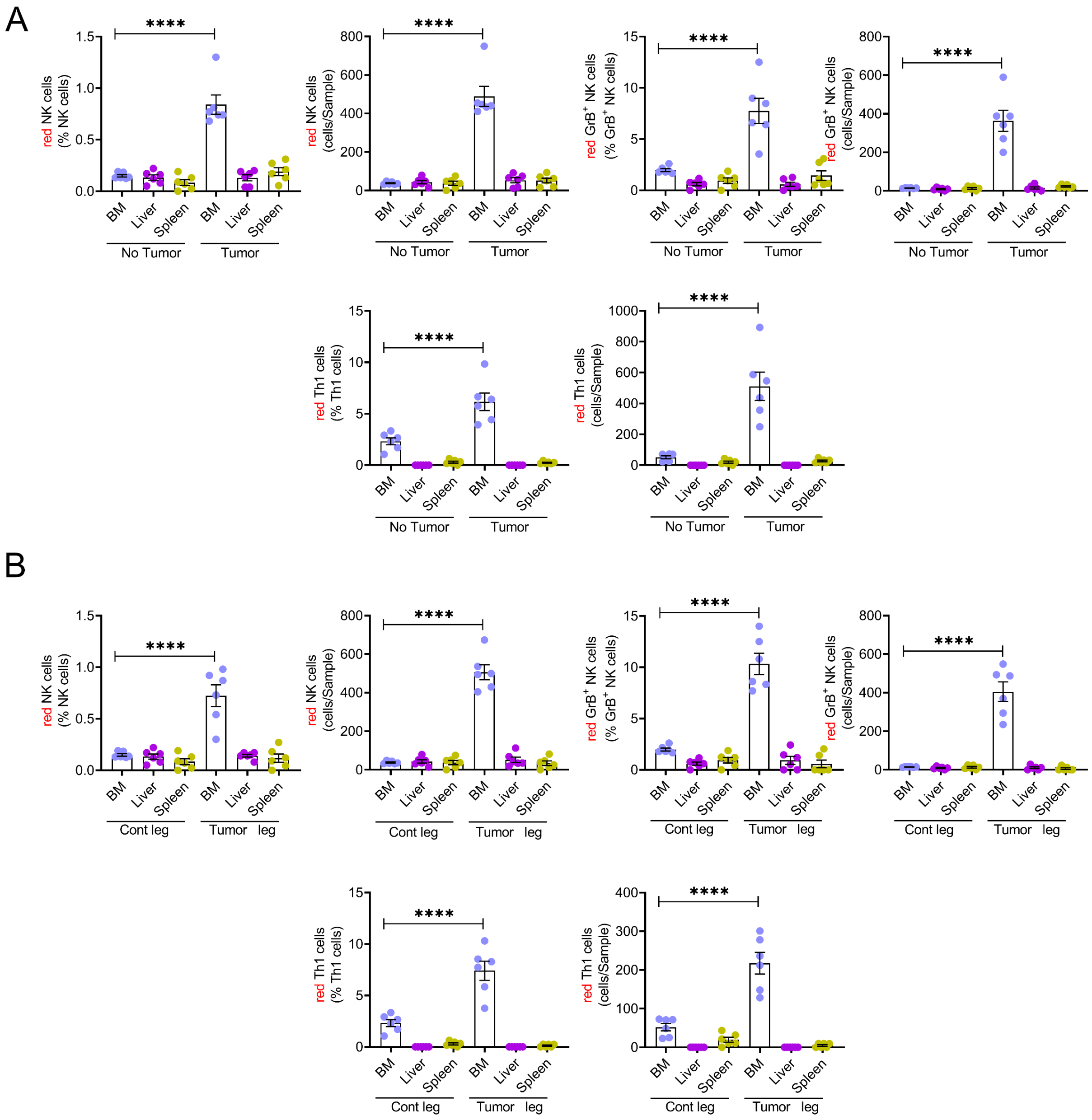

SF 10. Bone tumor growth specifically increases the trafficking of NK cells and Th1 cells from the gut to the BM. Intracardiac (panel A) or intratibial injections (panel B) of B16-F10 cells were carried out in 12-week-old female Kaede mice. 9 days later mice were subjected to surgical laparotomy to access the PPs in the distal SI. PP cells were photo-converted by exposing them to a 390 nm light for 2 minutes. Mice were sacrificed 24hr and 48hr after photoconversion. Relative frequency of red fluorescing NK ($NK1.1^+CD3^+$) cells, GrB^+ NK cells and Th1 ($CD3^+CD4^+IFN\gamma^+$) cells in the bone marrow (BM), liver and spleen. Data are expressed as Mean $\pm$ SEM. n= 6 mice per group. All data were normally distributed according to the Shapiro-Wilk normality test and analyzed by two-way analysis-of-variance and post hoc tests applying the Bonferroni correction for multiple comparisons. **** = $p<0.0001$ compared to the indicated group. Nonsignificant comparisons not shown.

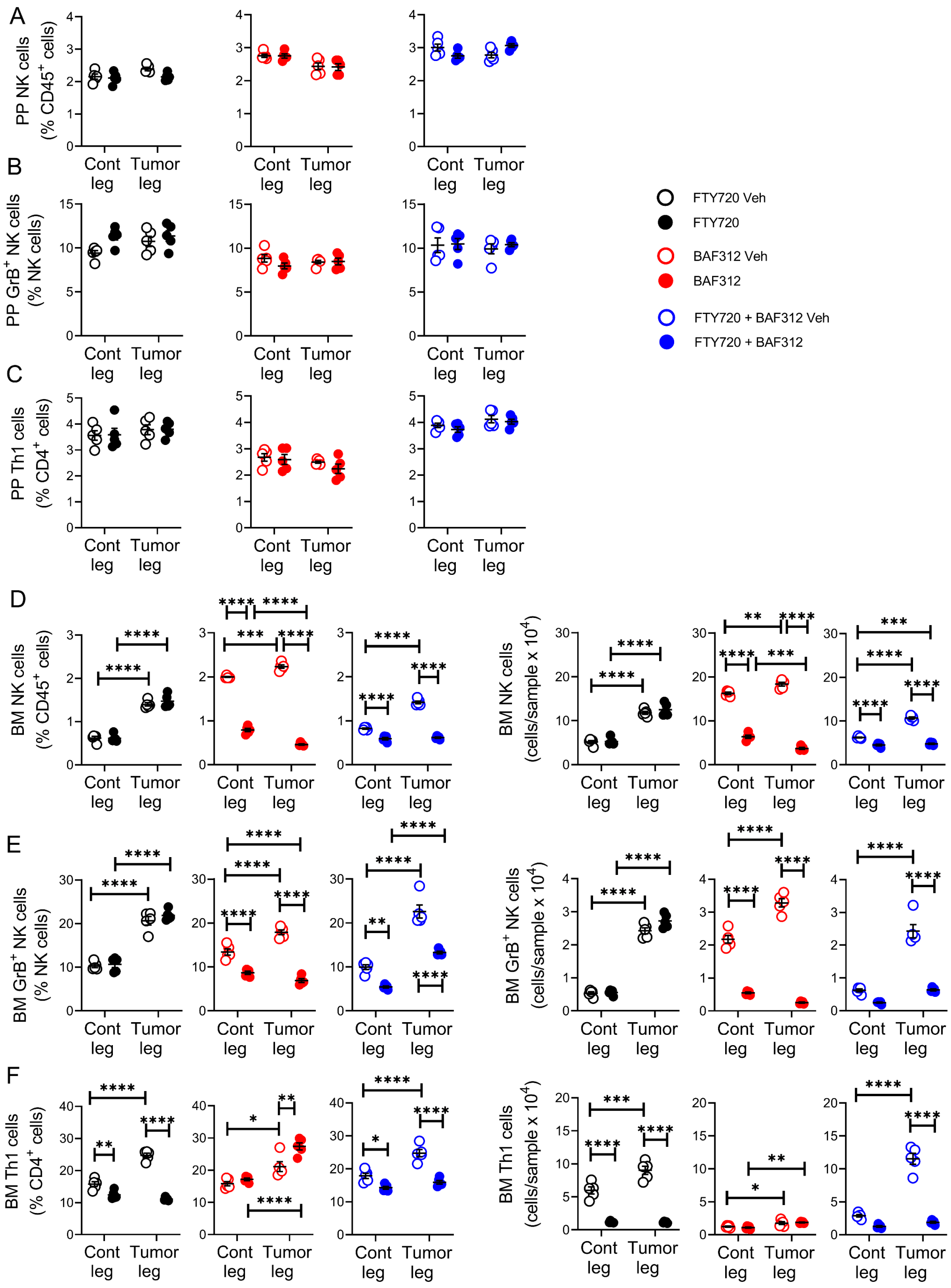

SF 11. Blockade of Th1 and NK cell egress from the gut prevents the expansion BM NK and Th1 cells induced by intratibial injections of melanoma cells. Intratibial injections of luciferase expressing B16-F10 melanoma cell line were carried out in 12-week-old C57BL/6 mice. The non-injected contralateral leg (Cont leg) was used as a control. Mice were also treated with the S1PR1 functional antagonist FTY720 and/or the S1PR5 functional antagonist BAF312, starting 1 week before the tumor cell injection. **A.** Relative frequency of PP NK ($NK1.1^+CD3^+$) cells. **B.** Relative frequency of PP GrB^+ cells. **C.** Relative frequency of PP Th1 ($CD3^+CD4^+IFN\gamma^+$) cells. **D.** Relative and absolute frequency of BM NK cells. **E.** Relative and absolute frequency of BM GrB^+ NK cells. **F.** Relative and absolute frequency of BM Th1 cells. n= 5 mice per group. Data are expressed as Mean $\pm$ SEM. All data were normally distributed according to the Shapiro-Wilk normality test and analyzed by two-way analysis-of-variance and post hoc tests applying the Bonferroni correction for multiple comparisons. *=p<0.05, **=p<0.01, ***=p<0.001 and ****= p<0.0001 compared to the indicated group. Nonsignificant comparisons not shown.

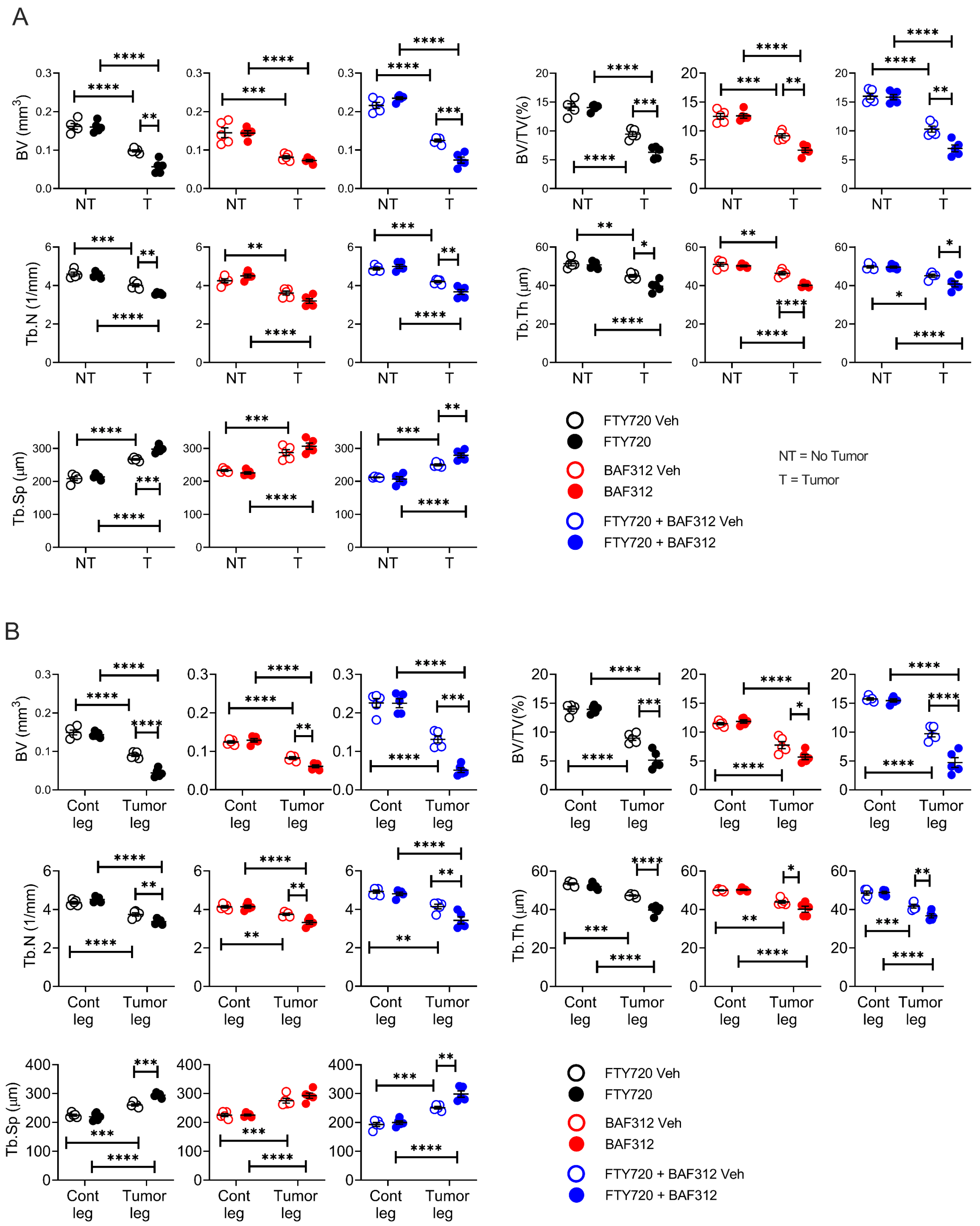

SF 12. Blockade of Th1 and NK cell egress from the gut worsens indices of trabecular structure caused by intracardiac and intratibial injections of melanoma cells. Intracardiac (Panel A) and intratibial (Panel B) injections of B16-F10 melanoma cell line were carried out in 12-week-old female C57BL/6 mice. In the intracardiac model, mice not injected with B16-F10 cells (No tumor) were used as controls. In the intratibial model, the non-injected contralateral leg (Cont leg) was used as control. Mice were also treated with the S1PR1 functional antagonist FTY720, and or the S1PR5 functional antagonist BAF312 starting 1 week before the tumor cell injection. n= 5 mice per group. Data are expressed as Mean ± SEM. All data were normally distributed according to the Shapiro-Wilk normality test and analyzed by two-way analysis-of-variance and post hoc tests applying the Bonferroni correction for multiple comparisons. *=p<0.05, **=p<0.01, ***=p<0.001 and ****= p<0.0001 compared to the indicated group. Nonsignificant comparisons not shown.

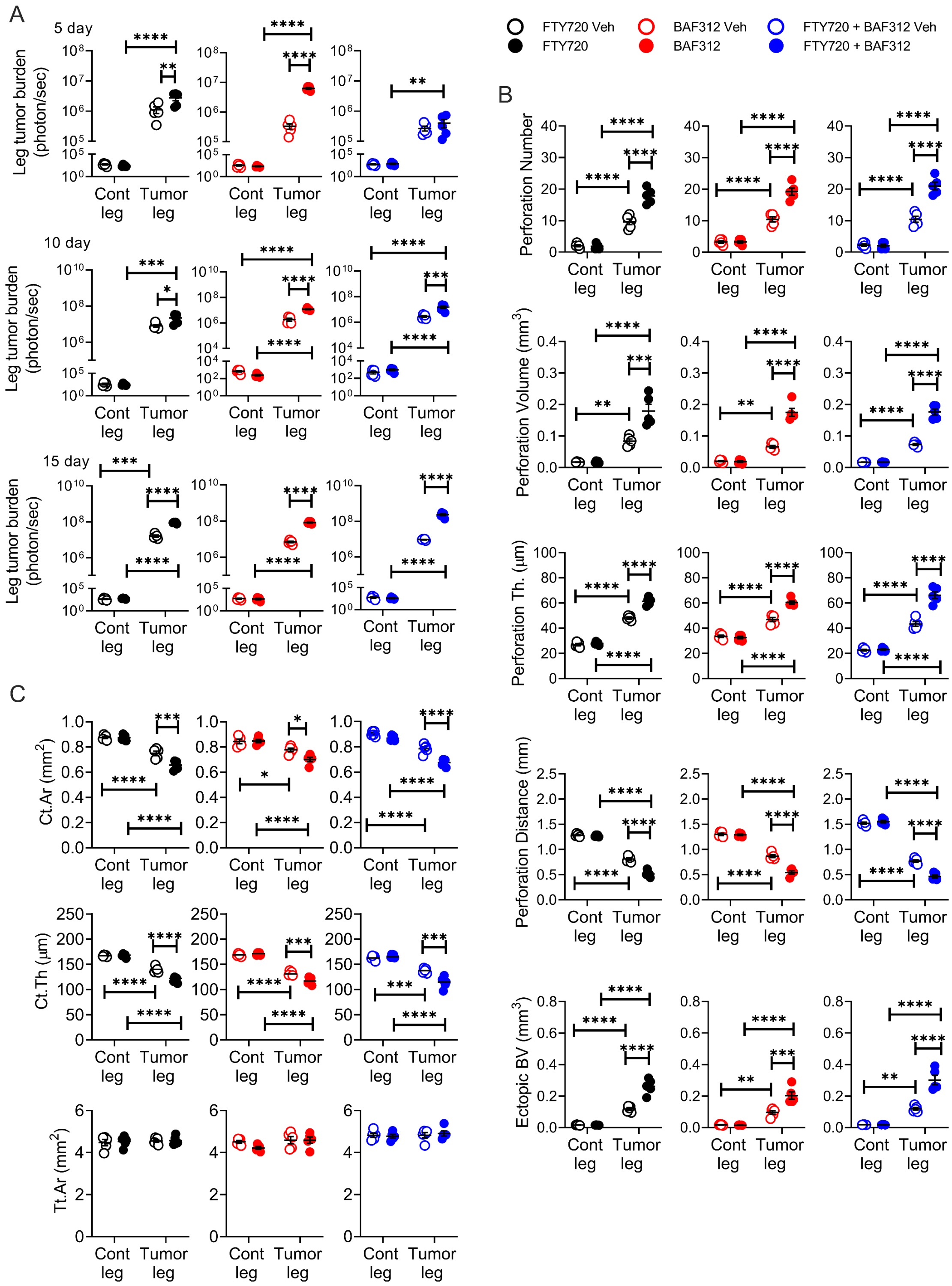

SF 13. Blockade of Th1 and NK cell egress from the gut accelerates bone tumor growth induced by intratibial injections of melanoma cells. Intratibial injections of luciferase expressing B16-F10 melanoma cell line were carried out in 12-week-old C57BL/6 mice. The non-injected contralateral leg (Cont leg) was used as a control. Mice were also treated with the S1PR1 functional antagonist FTY720 and/or the S1PR5 functional antagonist BAF312, starting 1 week before the tumor cell injection. NT = no Tumor. T = Tumor. **A.** Tumor growth as assessed by luminescence. **B.** Bone perforations and ectopic bone growth as assessed by µCT. **C.** µCT indices of cortical structure measured in tibial diaphysis. n= 5 mice per group. Data are expressed as Mean + SEM. All data were normally distributed according to the Shapiro-Wilk normality test and analyzed by two-way analysis-of-variance and post hoc tests applying the Bonferroni correction for multiple comparisons. *=p<0.05, **=p<0.01, ***=p<0.001 and ****= p<0.0001 compared to the indicated group. Nonsignificant comparisons not shown.

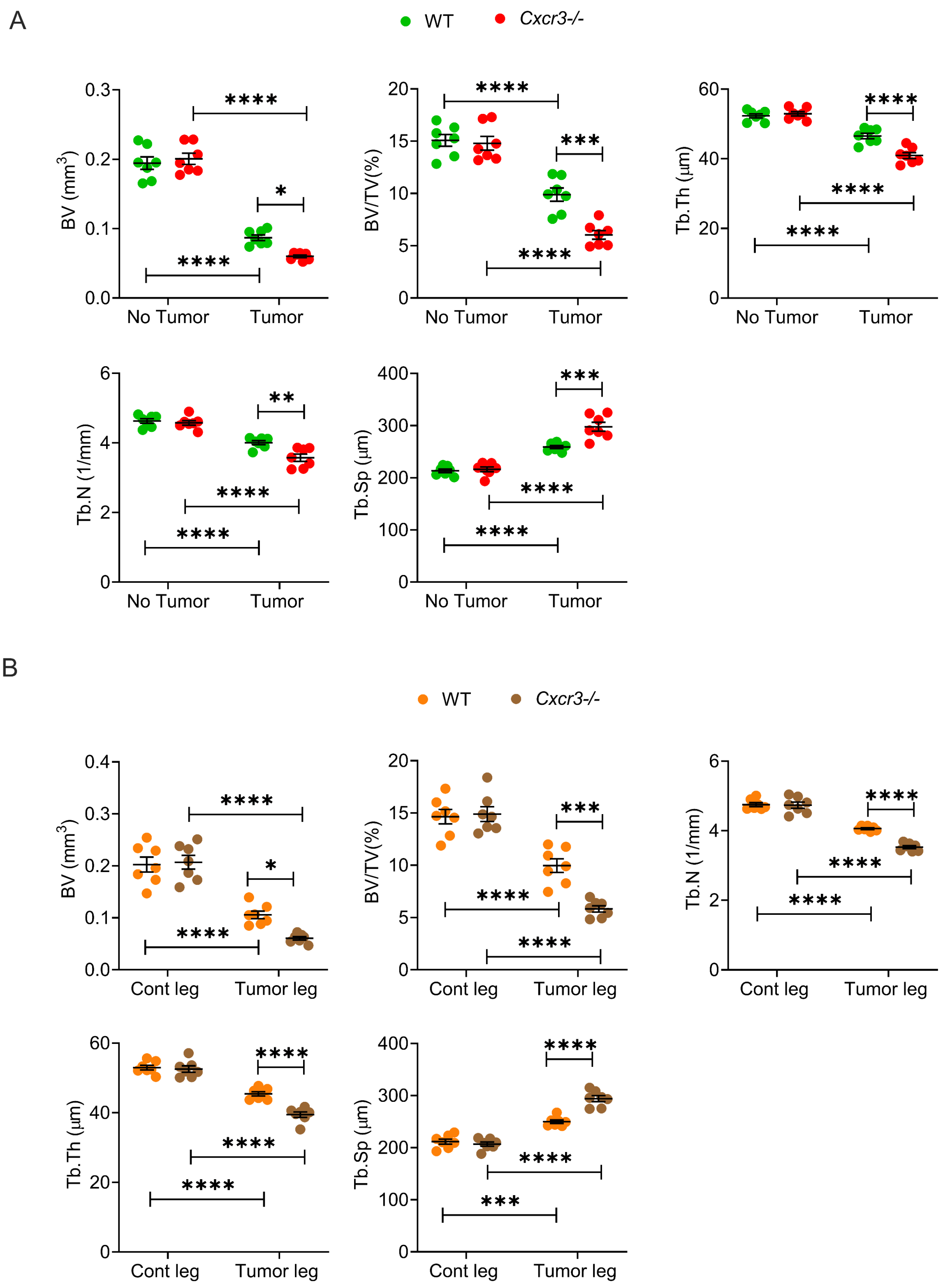

SF 14. Silencing of CXCR3 worsens indices of trabecular structure caused by intracardiac and intratibial injections of melanoma cells. Intracardiac (Panel A) and intratibial (Panel B) injections of B16-F10 melanoma cell line were carried out in 12-week-old female C57BL/6 WT and *Cxcr3-/-* mice. In the intracardiac model, mice not injected with B16-F10 cells (No tumor) were used as controls. In the intratibial model, the non-injected contralateral leg (Cont leg) was used as control. n= 7 mice per group. Data are expressed as Mean ± SEM. All data were normally distributed according to the Shapiro-Wilk normality test and analyzed by two-way analysis-of-variance and post hoc tests applying the Bonferroni correction for multiple comparisons. *=p<0.05, **=p<0.01, ***=p<0.001 and ****= p<0.0001 compared to the indicated group. Nonsignificant comparisons not shown.

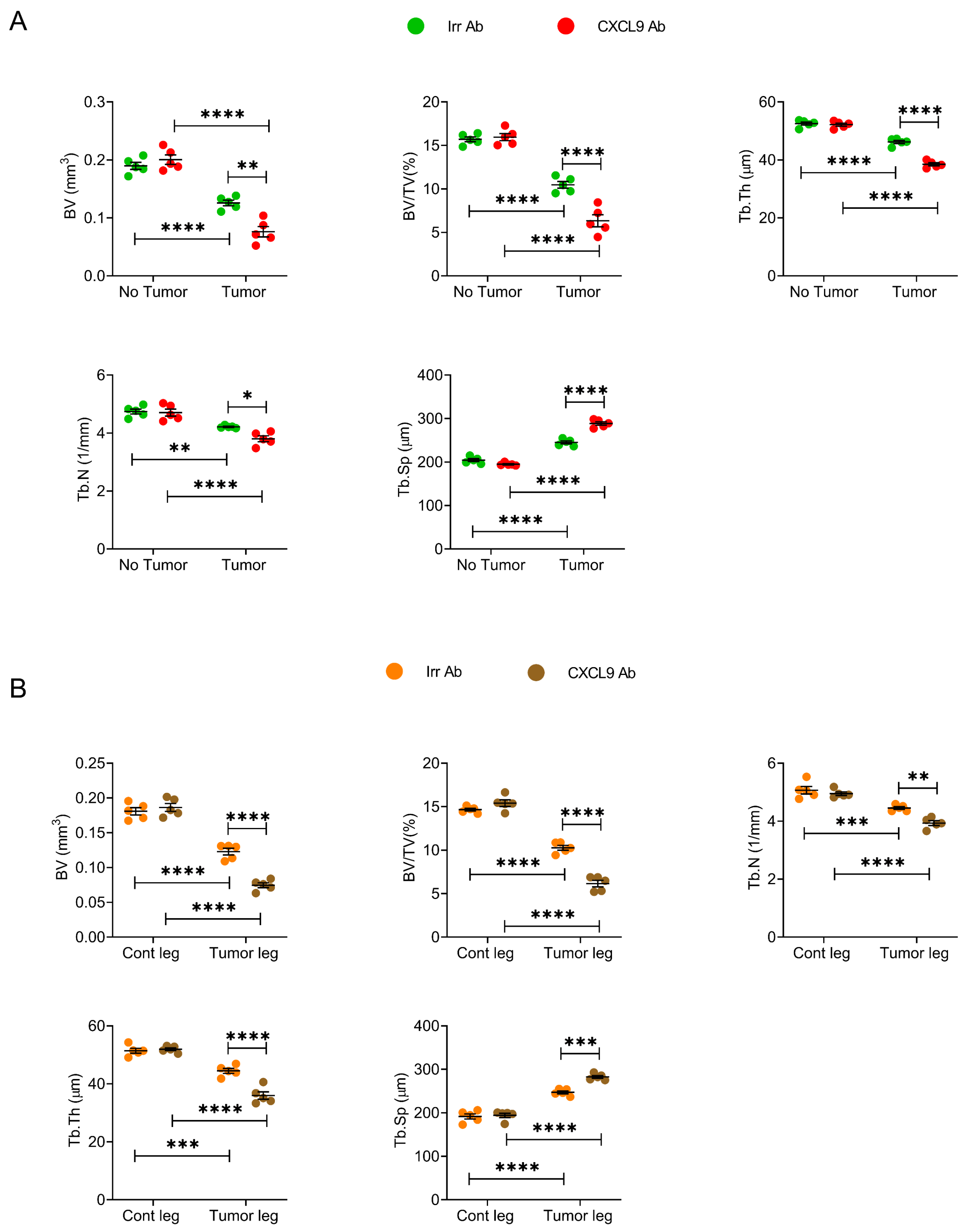

SF 15. Antibody neutralization of CXCL9 worsens indices of trabecular structure caused by intracardiac and intratibial injections of melanoma cells. Intracardiac (Panel A) and intratibial (Panel B) injections of B16-F10 melanoma cell line were carried out in 12-week-old female mice. In the intracardiac model, mice not injected with B16-F10 cells (No tumor) were used as controls. In the intratibial model, the non-injected contralateral leg (Cont leg) was used as control. Mice were treated with anti-CXCL9 antibody or isotype matched irrelevant antibody. n= 5 mice per group. Data are expressed as Mean $\pm$ SEM. All data were normally distributed according to the Shapiro-Wilk normality test and analyzed by two-way analysis-of-variance and post hoc tests applying the Bonferroni correction for multiple comparisons. *=p<0.05, **=p<0.01, ***=p<0.001 and ****= p<0.0001 compared to the indicated group. Nonsignificant comparisons not shown.

Supplemental table 1: List of the antibodies and reagents used for flow.

Name of the reagent	Catalog number
1. CD16/32 (clone 93)	Biolegend # 101302
2. BV 510-CD45 (clone 30-F11)	Biolegend # 103138
3. BV 421-TCRβ (clone H57-597)	Biolegend # 109230
4. AF 700-CD3 (clone 17A2)	Biolegend # 100216
5. AF 488-CD3 (clone 17A2)	Biolegend # 100210
6. PerCP/Cy5.5-CD4 (clone RM4-5)	Biolegend # 100540
7. BV 711-CD8 (clone 53-6.7)	Biolegend # 100748
8. PE-NK-1.1 (clone PK136)	Biolegend # 108708
9. BV 711-NK-1.1 (clone PK136)	Biolegend # 108745
10. AF488 -IFNγ (clone XMG1.2)	Biolegend # 505813
11. APC-IFNγ (clone XMG1.2)	Biolegend # 505810
12. AF 647-Granzyme B (clone GB11)	Biolegend # 515406
13. LIVE/DEAD Fixable Cell Stain Kit	ThermoFisher # L34959
14. Cell activation cocktail	Biolegend # 423301
15. Golgi blocker	Biolegend # 420701
16. Fixation & Permeabilization Buffer Set	Invitrogen # 88-8824-00
